# Ecological and Sociodemographic Determinants of House Infestation by *Triatoma infestans* in Indigenous Communities of the Argentine Chaco

**DOI:** 10.1371/journal.pntd.0003614

**Published:** 2015-03-18

**Authors:** M. Sol Gaspe, Yael M. Provecho, M. Victoria Cardinal, M. del Pilar Fernández, Ricardo E. Gürtler

**Affiliations:** Laboratory of Eco-Epidemiology, Department of Ecology, Genetics and Evolution, Universidad de Buenos Aires-IEGEBA (CONICET-UBA), Buenos Aires, Argentina; Centers for Disease Control and Prevention, UNITED STATES

## Abstract

**Background:**

The Gran Chaco ecoregion, a hotspot for Chagas and other neglected tropical diseases, is home to >20 indigenous peoples. Our objective was to identify the main ecological and sociodemographic determinants of house infestation and abundance of *Triatoma infestans* in traditional Qom populations including a Creole minority in Pampa del Indio, northeastern Argentina.

**Methods:**

A cross-sectional survey determined house infestation by timed-manual searches with a dislodging aerosol in 386 inhabited houses and administered questionnaires on selected variables before full-coverage insecticide spraying and annual vector surveillance. We fitted generalized linear models to two global models of domestic infestation and bug abundance, and estimated coefficients via multimodel inference with model averaging.

**Principal Findings:**

Most Qom households were larger and lived in small-sized, recently-built, precarious houses with fewer peridomestic structures, and fewer livestock and poultry than Creoles’. Qom households had lower educational level and unexpectedly high residential mobility. House infestation (31.9%) was much lower than expected from lack of recent insecticide spraying campaigns and was spatially aggregated. Nearly half of the infested houses examined had infected vectors. Qom households had higher prevalence of domestic infestation (29.2%) than Creoles’ (10.0%), although there is large uncertainty around the adjusted OR. Factors with high relative importance for domestic infestation and/or bug abundance were refuge availability, distance to the nearest infested house, domestic insecticide use, indoor presence of poultry, residential overcrowding, and household educational level.

**Conclusions and Significance:**

Our study highlights the importance of sociodemographic determinants of domestic infestation such as overcrowding, education and proximity to the nearest infested house, and corroborates the role of refuge availability, domestic use of insecticides and household size. These factors may be used for designing improved interventions for sustainable disease control and risk stratification. Housing instability, household mobility and migration patterns are key to understanding the process of house (re)infestation in the Gran Chaco.

## Author Summary

Indigenous peoples are especially affected by Chagas and other neglected tropical diseases. One of the most numerous ethnic groups in the Gran Chaco region of South America is the Qom (Toba) people. The living conditions of Qom households most likely differ substantially from those of Creoles, and their association with house infestation by triatomine bugs has not been investigated. This is important because the major vector species have adapted to thrive in human sleeping quarters, and in addition to housing construction patterns, other ecological and sociodemographic factors may affect house infestation. We found that Qom households had much higher domestic infestation than Creole ones, in conjunction with more precarious housing, fewer poultry and livestock. The unexpectedly high local residential mobility of Qom households combined with the large fraction of recently-built houses (derived from a rapidly increasing population with a very young age structure during recent decades) may explain why domestic infestation was much lower than expected from the lack of recent insecticide spraying campaigns. Domestic infestation and bug abundance increased with overcrowding and refuge availability, and decreased with household education levels and insecticide use. These results are useful for designing improved interventions and household risk stratification.

## Introduction

The strong association between neglected tropical diseases (NTDs), poverty and particular combinations of ecological, social, political and economic determinants explains the occurrence of global hotspots of NTDs [1]. One of such hotspots occurs in the Gran Chaco ecoregion in South America, where the prevalence rates of geohelminthic infections and Chagas disease are very high [1]. Chagas disease, caused by *Trypanosoma cruzi*, is considered the main regional vector-borne disease in terms of disease burden and affects 8–10 million people in Latin America [2]. *Triatoma infestans*, the main vector in the Southern Cone countries and southern Peru, has been the target of an insecticide-based regional elimination program that interrupted the transmission of human *T*. *cruzi* infection by *T*. *infestans* in various countries [2–4]. Progress in the Gran Chaco lagged behind and vector-mediated transmission of *T*. *cruzi* still occurs albeit at lower incidence levels than 20 years ago [5–8].

The Gran Chaco is home to more than 20 ethnic groups [9]. Indigenous populations usually are among the most marginalized groups, with more precarious health and living conditions than other peoples [10–12]. Indigenous communities of the Gran Chaco showed high seroprevalence of human *T*. *cruzi* infection [13–18]. One of the most numerous ethnic groups in this region is the Qom (Toba) people [19]. Qom households were exposed to a greater risk of *T*. *cruzi* infection than Creole households in a well-defined rural section of Pampa del Indio (Argentine Chaco) mainly inhabited by Creoles (denominated Area I), but there were large heterogeneities between and within ethnic groups [20,21]. Further studies on the ecological, biological and social (eco-bio-social) determinants of vector-borne diseases are needed [22], more so in the case of vulnerable indigenous populations affected by Chagas disease and other NTDs.

The main identified determinants of house infestation with the major domestic vectors of *T*. *cruzi* (*T*. *infestans*, *Rhodnius prolixus*, *Panstrongylus megistus*, and *Triatoma dimidiata*) include housing construction characteristics that create refuges for the bugs to hide in (e.g., cracks in walls, thatched roofs, precarious peridomestic structures); the presence and number of human and domestic animal hosts (dogs, chickens) that serve as bloodmeal sources, and little or no domestic application of insecticides by house residents [20,23–32]. These factors are the expression of various underlying processes that ultimately create conditions that facilitate house infestation and *T*. *cruzi* transmission [33]. A full understanding of complex systems [34] involving infectious diseases requires more integrative approaches such as the ecosystem approach to human health (ecohealth) [35], which gives proper attention to eco-bio-social factors and their eventual interactions. However, very few studies have explicitly addressed these factors simultaneously in relation to Chagas disease [31,36,37]. This limited knowledge curtails our ability to design and implement innovative vector and disease control strategies adapted to resource-constrained settings. The current study therefore addressed traditional ecological determinants and selected sociodemographic factors related to poverty and ethnicity.

As part of a longitudinal study on the eco-epidemiology and control of Chagas disease in northeastern Argentina, we expanded the scope and geographic scale of our previous studies [20,21,37] conducted in Area I of Pampa del Indio to focus on Qom communities living in ancestral territories which also included a Creole minority (denominated Area III). The living conditions of Qom households most likely differed substantially from those of Creoles, and their association with house infestation has not been investigated at a sufficiently large spatial scale. The objective of the current study was to identify the main ecological and sociodemographic determinants of domestic infestation and abundance of *T*. *infestans* (two surrogate indices for transmission risk) in Area III, where Qom communities predominated, using generalized linear models in a multimodel inference frame with model averaging. In addition to the above-mentioned factors known to be closely associated with house infestation in multiple settings, we examined the effects of distance to the nearest infested house, residential overcrowding, household education level, wealth indicators, and preventive practices. The first two factors were predicted to exert positive effects on domestic infestation and bug abundance whereas the remaining factors were expected to exert negative effects. We also re-examined whether ethnic background modified both response variables when other relevant risk factors were accounted for. Our study highlights the relevance of various ecological and sociodemographic factors whose effects have not been investigated simultaneously, and provides guidance on improved control interventions specifically adapted to the Gran Chaco.

## Materials and Methods

### Study area

**Fig 1:**
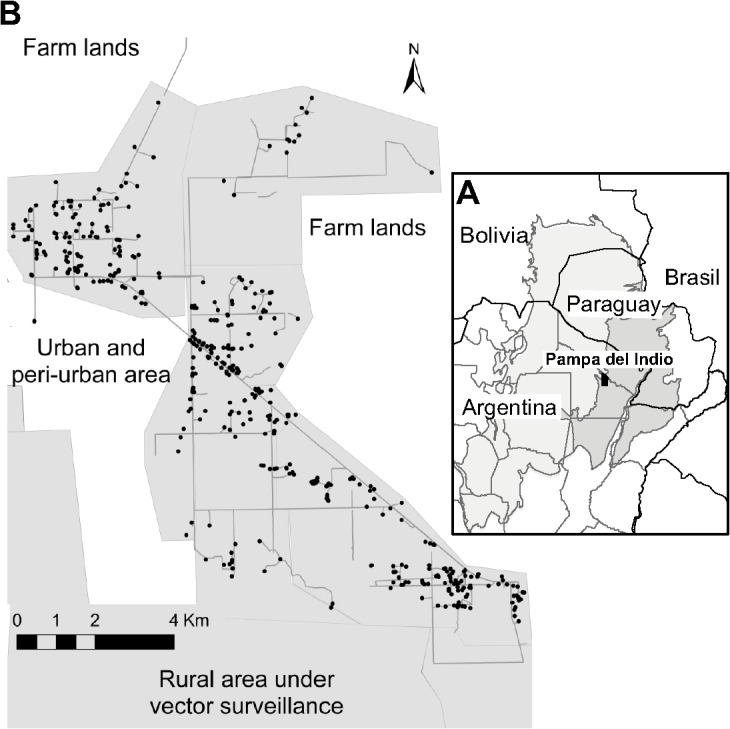
Location of the humid (dark gray) and dry (light gray) Gran Chaco region (A) and map of the study area (B). Pampa del Indio, Chaco, October 2008. Map shows the houses surveyed (dots) and surrounding areas.

Field work was conducted in a rural section (95 km^2^) of Pampa del Indio municipality (25° 55’S 56° 58’W), Chaco province, Argentina (Fig. 1). The municipality was inhabited by approximately 22,000 people by late 2013, and 45% of residents belonged to the Qom ethnic group according to local municipal authorities. Official decennial census records in 2001 and 2010 indicated that the population of Pampa del Indio municipality increased remarkably from 11,558 to about 18,000 people, respectively (annual population growth rate, 4.9%). The climate, landscape and demographic features of a contiguous section of the municipality inhabited mainly by Creole households were described elsewhere [20,21]. The last insecticide spraying campaign conducted in the municipality occurred in 1997–1998 according to the Chagas disease control program from Chaco province. Selection of the study area took into account the lack of recent history of community-wide insecticide spraying; preliminary evidence of house infestation ranging from 30 to 40%; the predominance of indigenous households; and the presence of at least 350 adjacent households in order to achieve a sufficiently large study base for statistical inference.

A household is defined as all the people who occupy a housing unit including the related and nonrelated family members [38]. A house compound was defined as the set of domicile (i.e., an independent structure used as human sleeping quarters, S1 Fig.), patio and other structures included within the peridomestic area (kitchens, storerooms, latrines, corrals, chicken coops and chicken nests (“nidero”), ovens, trees where chickens roosted, others) as illustrated elsewhere [20]. House compounds sometimes had more than one domicile used as sleeping quarters by related family (S1 Fig.).

### Study design and vector survey

Before initiating field operations local key actors were interviewed to gather background data that may allow a better assessment of the preintervention situation; discuss the initial and long-term goals of the research program (see below); and assist the interpretation of the study outcomes. Local key actors included the mayor, health and education authorities and other personnel, rural health-care workers and school teachers, representatives of third-sector organizations, and community leaders. The stated long-term goals of the research program were to interrupt the human transmission of *T*. *cruzi* through intensified vector control, human diagnosis and treatment, and to promote long-term sustainability of disease control efforts through local empowerment.

A cross-sectional survey aimed at enumerating all house compounds in the area and assessing house infestation was conducted in October 14–31, 2008. The study area included seven villages with 407 inhabited houses, 19 abandoned dwellings and 17 public buildings (4 primary schools, 1 health-care post, 6 churches and 6 community centers) (Fig. 1). One member of the research team explained to each householder the aims of the survey and requested permission to access their premises and identify the house with a numbered aluminum plate. The location of each house was georeferenced with a GPS receiver (Garmin Legend). Householders were asked for the presence of triatomines within their premises after showing them dry specimens of *T*. *infestans*, *Triatoma sordida* and other Reduviidae to prevent confusion with other insects; from these reports we derived the index “householders’ notification of house infestation”. All households were provided with a labeled self-sealing plastic bag to contain any triatomine they sighted, and instructed on how to manipulate the bugs safely. This additional source of bugs was denominated “householders’ bug collections”. Householders’ bug collections were only considered if the date and collection site were reported to us. The study protocol was approved by the Dr. Carlos A. Barclay Independent Ethical Committee for Clinical Research, Buenos Aires, Argentina.

A total of 386 inhabited houses (94.9%) were included in the current study of triatomine infestation; 21 houses closed during the survey were not searched for bugs. In all of the 386 study houses the following methods were performed to assess bug infestation: i) inspection by timed-manual searches; ii) collection of bugs that were spotted during insecticide spraying operations; and iii) promotion of householders’ collection of any triatomine they sighted (as explained above). Multiple methods were used as a cross-check of the outcome of timed-manual searches. All domestic and peridomestic sites of the study houses were searched for triatomine bugs (timed-manual collections) by four teams including one supervisor and two or three skilled bug collectors who used 0.2% tetramethrin (Espacial, Argentina) to dislodge the insects as described [20]. Each domicile and peridomestic site was searched by one person for 15 min. Immediately after the vector survey, vector control personnel sprayed every house with suspension concentrate deltamethrin (K-Othrin, Bayer) or beta-cypermethrin (Sipertrin, Chemotecnica) using standard doses (25 and 50 mg/m^2^, respectively) and routine procedures [39]. Bugs sighted during insecticide spraying operations were also collected.

The collected bugs were stored in plastic bags labeled with the house number and specific bug collection site and were transported to the field laboratory where they were identified taxonomically and counted according to species, stage or sex. Two to six weeks after bug collection, feces of all the third-instar nymphs and older stages that were alive were microscopically examined for infection with *T*. *cruzi* at 400× as described [21]. The bugs examined for infection were collected from 72.8% of the infested houses.

### Environmental and sociodemographic survey

This survey was conducted in parallel to the vector survey in October 2008. An adult household member fluent in Spanish was asked for information on the following items: full name of householder (i.e., head of household) [40]; the number of resident people by age class (0–5, 6–14, and 15 or more years of age); the number of domestic animals of each type (dogs, cats, chickens, other poultry, goats, pigs, cows, and equines) and their resting places; use of domestic insecticides (type, frequency, purpose); and date of the last insecticide spraying of house premises conducted by vector control personnel or the local hospital or any other third party using manual compression sprayers. Because the study area encompassed traditional Qom communities, assignment of a household to ethnic group was based on whether they spoke Qom language (Qomlaqtaq); participated in traditional Qom organizations; and took into account their physical features. When in doubt, assignments to ethnic group were subsequently checked with local Qom health-care personnel and were corroborated in all cases. Households with a mixed ethnic background were considered to be Qom because they resided in ancestral indigenous territories and fulfilled the above mentioned attributes.

A sketch map of the spatial location of all structures in each house compound was performed, and each structure was given a unique code according to its use. We recorded the building materials used in roofs and walls, presence of wall plaster, condition of wall surface, and plaster material. The availability of refuges for bugs was determined visually by a skilled member of the research team and scored in one of five levels ranging from absence to very abundant refuges [20]; only the three top categories were recorded in domiciles.

As our knowledge of the study area increased during the vector surveillance phase, additional sociodemographic variables potentially associated with house infestation were taken into consideration and recorded mostly in November 2012: educational level attained by each household member (number of schooling years completed); land ownership (*no ownership*; *individual*: the householder owned the land they inhabited; *familial*: a relative owned the land; *communal*: the community owned the land which therefore could not be sold); agricultural activities (present and past); monthly public welfare support; household electricity and time since first connection; age of house (years since construction); size of each domicile’s area; source of drinking water; presence of window screens (wire mesh); use of bed nets; and participation in a local social organization.

The data collected in 2012 were back-corrected to extant conditions in 2008 (e.g., access to electricity, age of house, agricultural activities). Overall changes in several respects (e.g., drinking water source, domicile’s area, participation in social organizations) were negligible during the four-year period. For some of the back-corrected variables it was possible to assess the validity of the reports. The comparison of domestic area and age of house recorded both in 2009 and 2012 showed minor differences. Land tenure, access to electricity and householders’ reports of time since last insecticide spraying were checked with other local sources of information and whether they were spatially clustered. Comparison between the list of houses sprayed with insecticides in 2006 (identified by the name of the head of household) and the date of the last insecticide spraying each individual household reported to us in 2008 showed either large or perfect agreement in two communities (75% and 100%) and a very low degree of agreement in another community (8%).

The recorded data were used to compute household-level surrogate indices for wealth, educational level and overcrowding. The goat-equivalent index represents a small stock unit to quantify the total number of livestock (cows, pigs, goats) and poultry owned by the household in terms of goat biomass. To calculate this index the average weight of each type of animal was considered (cow, 453 kg; pig, 159 kg; goat, 49 kg; chicken, 2.5 kg) [41]. The household educational level was defined as the mean number of schooling years attained by household members aged 15 years old or more [42]. The overcrowding index was defined as the number of human occupants per sleeping quarter; the presence of 3 or more occupants per room is taken as critical overcrowding [43]. Housing quality (a three-level categorical variable) was represented by the combination of mud walls (versus brick-cement walls) and tarred-cardboard sheets on the roof (versus corrugated metal-sheets); no house had brick-cement walls and tarred-cardboard sheets.

As part of annual vector surveillance after community-wide insecticide spraying in October 2008, all extant households in the study area were re-surveyed in August 2009, April 2010 and November 2012, whereas a sample of 86 houses was re-surveyed in December 2011. For each house we recorded its current and previous existence; fate (destruction, movement and construction); destination of moving households and underlying reasons (whenever possible), among other variables. The sociodemographic information was collected at every new house as in the baseline survey.

### Data analysis

House infestation data only included inhabited houses because no local public building or abandoned house was found to be infested. Similarly, latrines and trees used by chickens were not infested by *T*. *infestans* and therefore were not included in the number of peridomestic sites per household. The prevalence of house (or site-specific) infestation by *T*. *infestans* was calculated based on the finding of at least one live bug (except eggs) by any of the bug collection methods used (i.e., timed-manual searches, during insecticide spraying operations, and householders’ bug collections) relative to the total number of houses (or sites for each ecotope) inspected. The abundance of triatomine bugs was calculated as the number of live bugs collected per 15 min-person among houses positive by timed-manual searches. If a house compound had more than one domicile, the average domestic bug abundance was calculated as the total number of live triatomines collected per 15 min-person across domiciles divided by the number of domiciles inspected. A matrix of distances to the nearest infested house was calculated using qGIS [44]. Agresti–Coull binomial 95% confidence intervals (95% CIs) were used for infestation prevalence [45].

Householders’ notification of the domestic presence of *T*. *infestans* and timed searches of domestic infestation were compared using the kappa index implemented in Stata 12 [46]. Kappa index values greater than 0.6 may be considered substantial to perfect agreement and values less than 0.4 represent a poor agreement beyond chance.

Risk factor analyses of the presence and relative abundance of *T*. *infestans* were restricted to human sleeping quarters because peridomestic infestations were relatively few. Owing to the occurrence of house compounds with more than one domicile (including related family) and that several variables were measured at the household level, in these cases data for all domiciles were pooled to obtain a single observation per compound. Availability of refuges for bugs and age of house were averaged over domiciles within a house compound, and the total domestic area was the sum of each domicile’s area. The number of domestic hosts (dogs or cats and poultry, mostly chickens) used in the analyses (not in the census) only included animals reported to rest or nest inside domiciles. Bivariate logistic and negative binomial regressions on each explanatory variable were performed with domestic infestation and bug abundance as response variables, respectively. Relative bug abundance (RA), labeled in Stata output as 'incidence-rate ratios’, and their CIs were calculated from the estimated coefficients (b) of the negative binomial regression as e^b^.

### Multivariate analysis

The association between selected explanatory variables and both response variables were tested through multiple logistic and negative binomial regressions, respectively. The global models included 10–12 explanatory variables with complete data selected a priori based on background evidence (e.g., [20,25,30]) and additional hypothesis on the predicted effects of selected sociodemographic determinants as mentioned above. Some variables measured in 2012 (i.e., age of house, electricity, time since last insecticide spraying) had a large number of missing data and therefore were not included in these analyses. We also compared the fit of the negative binomial models for bug abundance with those returned by mixture and two-part models for zero-inflated distributions, and found strong evidence of the superiority of the negative binomial regression model (S2 Text).

Two global models were analyzed. The first model included 10 explanatory variables (from 386 households) which described building characteristics (housing quality, refuge availability), domestic host availability (number of persons, number of dogs or cats and presence of poultry indoors), socioeconomic features (ethnicity, goat-equivalent index), household preventive practices (reported insecticide use), peridomestic infestation by *T*. *infestans*, and distance to the nearest infested house. The second model included 12 variables (i.e., the 10 variables mentioned before, residential overcrowding and household educational level) recorded at 274 households. Some continuous variables were rescaled in order to give more meaning to the unit of increment of risk estimates: distance to the nearest infested house (one unit every 50 m), household educational level (every 6 years) and the goat-equivalent index (every 10 goats). For comparative purposes we also analyzed the second data set after removing overcrowding and educational level data. On a post hoc basis we investigated the effects of the interactions between ethnicity and every other factor in the global models on both response variables, which proved not to be significant. These terms were added one by one to each global model and tested separately to avoid convergence problems.

Potential multicollinearity among explanatory variables was evaluated through the variance inflation factor (VIF) and condition numbers as implemented in Stata 12. The condition numbers were less than 10 and VIF < 2 for all explanatory variables, indicating that the significant correlation found between some pairs of variables (ethnic group with housing quality, refuge availability, goat-equivalents, which had correlation coefficients ranging from 0.35 to 0.4) would not cause serious multicollinearity.

We used an information theoretic approach and Akaike’s information criterion (AIC) to identify the best-fitting models describing variations in domestic infestation and abundance of *T*. *infestans*, given the data collected. Multimodel inference was especially conceived to account for model selection uncertainty; it allows a quantitative ranking of the models and identification of the set of models having best support given the data [47,48]. Because the ratio between the number of parameters and the number of observations (i.e., houses) was less than 40, we used the AIC corrected for small sample size (AIC_c_). Akaike differences (ΔAIC_c_) were calculated for each model as ΔAIC_c_ = AIC_c_—AIC_min_; models with ΔAIC_c_ > 2 were considered to have less support than the best model (AIC_min_), given the data and models analyzed. Several models had substantial support; therefore, we performed multimodel inference through model averaging. The Akaike weight (w_i_) of a model represents the support or probability of being the “best model”. The relative importance (RI) of each variable is defined as the sum of Akaike weights in each model in which the variable is present; RI takes values from 0 to 1. The overall fit of the logistic models was assessed by the Hosmer-Lemeshow test using the model-averaged coefficients and pooling the data in 10 equal-sized groups. Odds Ratios (ORs) and their 95% confidence intervals were calculated from model-averaged coefficients. Unconditional standard errors were calculated according to equation 4 in [49] with the default option (revised.var = TRUE). The area under the receiver operating curve (ROC) was also calculated; a value of 1 indicates a perfect fit. Sensitivity and specificity were assessed using the observed infestation prevalence of each data set as the cutoff values. The analyses and calculations were performed in R (version 2.15.1) [50]. Package MuMIn (version 1.9.5) was used for multimodel averaging; ResourceSelection (0.2–2) for performing the Hosmer-Lemeshow test; and ROCR (version 1.0–5) for calculating sensitivity, specificity and the area under the ROC curve.

### Spatial analysis

The spatial distribution of domestic infestation was assessed through global and local point pattern analyses (PPA) [51]. The former estimates the spatial aggregation of the outcome event across the entire study area whereas the latter detects the location of aggregated events. The spatial distribution of houses was examined to determine whether the potential aggregation of house infestation was influenced by a non-random dispersion of house locations.

The global spatial analysis of domestic infestation was performed in *Programita* using the weighted *K-function* [52] and random labeling as the null hypothesis (i.e., to assess the spatial distribution of infested houses given the fixed spatial distribution of all houses). The maximum distance considered was 2,000 m (i.e., one-third of the smallest dimension of the area) [51], and the cell size was 40 m. A total of 999 Monte Carlo simulations was performed and the 95% confidence envelope was calculated with the 25th upper and lower simulations.

Local spatial aggregation of infestation was tested through the Getis statistic (G*) [53] implemented in PPA [54]. This analysis distinguishes between positive and negative aggregation of events (i.e., infested houses); parameter settings were the same as for the global analysis.

## Results

### Population characteristics

The house-to-house census enumerated a total of 2,389 inhabitants in 386 inhabited houses as of October 2008, and 2,356 persons in 445 inhabited households in November 2012. The population included 18.0% up to five years of age; 27.7% between 6 and 14 years; and 54.3% with 15 or more years of age at baseline, and displayed a nearly indistinguishable age distribution in 2012. The mean age was 20.3 yr whereas the country-wide average was 34.4 yr. The number of men per 100 women was 109.2 whereas the average for Argentina was 95.8 as of 2010 [55].

A summary of the housing and sociodemographic characteristics of the study population by ethnic group is shown in Table 1. The detailed frequency distribution of study variables appears in Tables 2, 3 and S1. Qom households comprised 89.6% of the inhabited houses. Unlike Creoles, most Qom households lived in houses with mud walls and a tarred-cardboard roof, in small-sized (< 30m^2^), recently-built domiciles with high refuge availability, < 2 peridomestic structures, and with little access to electricity (Table 1). Qom households were larger, more often experienced critical overcrowding (S1 Text), and had lower household educational level than Creoles. The average goat-equivalent index of Creoles (median, 68.9; first-third quartiles, 5.7–168.6) was 69 times larger than that of Qom households (1.0, 0.1–7.7).

**Table 1:** Summary of housing and sociodemographic characteristics by ethnic group, Pampa del Indio, Chaco.

Variables	% of households (no. of houses)		
Housing characteristics	Qom	Creole	Total
Mud walls	84.1 (346)	37.5 (40)	79.3 (386)
Cardboard roofs	58.4 (346)	17.5 (40)	54.2 (386)
Low housing quality^a^	55.2 (346)	12.5 (40)	50.8 (386)
High refuge availability^b^	69.9 (346)	37.5 (40)	66.3 (386)
Recently built^c^	49.2 (250)	25.7 (35)	46.3 (285)
Small domestic area^d^	44.3 (300)	23.7 (38)	42.0 (338)
Electricity	22.2 (225)	58.8 (34)	27.0 (259)
Few or none peridomestic structures^e^	54.3 (346)	12.5 (40)	50.0 (384)
Sociodemographic characteristics			
Large household size^f^	69.3 (346)	32.5 (40)	65.5 (386)
Critical overcrowding^g^	56.3 (238)	15.8 (38)	50.7 (276)
Low household educational level^h^	60.7 (257)	51.3 (39)	59.5 (296)
Insecticide use	42.8 (346)	90.0 (40)	47.8 (386)
Window screen	7.3 (233)	59.5 (37)	14.4 (270)
Agricultural activities	85.5 (166)	79.3 (29)	84.6 (195)
High goat-equivalent index^i^	12.7 (346)	65.0 (40)	18.1(386)
Public welfare support	71.3 (240)	54.3 (35)	69.1 (275)

^a^ mud walls and cardboard roof.

^b^ categories 4 and 5 of the index.

^c^ less than 5 years.

^d^ less than 30 m^2^ (median domestic area).

^e^ less than 2 peridomestic structures (excluding latrines and trees with chickens).

^f^ more than 4 residents.

^g^ 3 or more residents per sleeping quarter.

^h^ less than 6 years of schooling among household residents aged >15 years old.

^i^ more than 30 goat-equivalents.

Households with missing data were excluded from each variable. Age of house, domestic area, electricity, number of rooms, household educational level, window screens, agricultural activities and public welfare support were recorded in 2012.

**Table 2:** Distribution of domestic infestation prevalence and abundance of *T. infestans* according to housing, host and other characteristics, Pampa del Indio, Chaco, October 2008.

Variable		Infestation prevalence^a^ (no. of inspected houses, % of total)	OR (CI)†	Median bug abundance^b^ (1st-3rd quartiles) (no. of infested houses)	RA (CI)†
Mud walls					
	Yes	30.4 (306, 79.3)	2.5 (1.3; 4.8)*	3.0 (1–11) (78)	2.1 (0.8–5.3)
	No	15.0 (80, 20.7)	1	1.5 (1–4) (10)	-
Cardboard roof					
	Yes	33.0 (209, 54.1)	1.9 (1.2; 3.1)*	2.0 (1–9) (58)	1.4 (0.7–3.0)
	No	20.3 (177, 45.9)	1	2.5 (1–12) (30)	-
Age of house (years)					
	≤ 1	29.4 (34, 11.9)	1	4.0 (2–7) (8)	-
	2–5	31.6 (98, 34.4)	1.1 (0.2; 1.8)	3.0 (1–12) (30)	2.5 (0.6–10.5)
	6–10	24.7 (73, 25.6)	0.8 (0.3; 2.0)	2.0 (1–4) (13)	1.1 (0.2–4.8)
	11–20	22.9 (48, 16.9)	0.7 (0.3; 1.9)	7.5 (3–13) (8)	1.2 (0.2–6.0)
	> 20	18.9 (32, 11.2)	0.6 (0.2; 1.8)	1.0 (1–3) (5)	0.4 (0.1–2.5)
Refuge availability					
	3	14.0 (129, 33.4)	1	1.0 (1–2) (13)	-
	4	27.6 (152, 39.4)	2.4 (1.3; 4.3)*	2.0 (1–9) (38)	13.8 (5.8–32.7)*
	5	42.9 (105, 27.2)	4.6 (2.5; 8.7)*	4.0 (1–15) (37)	27.5 (10.9–69.4)*
Domestic area (m^2^)					
	0–10	50.0 (14, 4.1)	1	7.0 (2–15) (7)	-
	11–30	32.8 (128, 37.9)	0.5 (0.2; 1.5)	2.0 (1–11) (36)	0.2 (0.03–1.6)
	31–50	28.1 (96, 28.4)	0.4 (0.1; 1.2)	2.0 (1–9) (23)	0.2 (0.02–1.2)
	51–100	18.2 (77, 22.8)	0.2 (0.1; 0.7)*	5.0 (2–11) (10)	0.1 (0.01–0.7)*
	> 100	13.0 (23, 6.8)	0.2 (0.0; 0.7)*	30.5 (3–58) (2)	0.2 (0.02–2.7)
No. of people					
	1–2	18.0 (61, 15.8)	1	2.0 (1–7) (10)	-
	3–6	27.1 (155, 40.2)	1.7 (0.8; 3.6)	1.5 (1–3) (34)	0.5 (0.2–1.5)
	7–10	28.0 (132, 34.2)	1.8 (0.8; 3.8)	4.0 (2–12) (33)	1.5 (0.5–4.6)
	>10	39.5 (38, 9.8)	3.0 (1.2; 7.5)*	4.0 (1–13) (11)	1.5 (0.3–6.4)
No. of poultry indoors^c^					
	0	24.8 (298, 77.2)	1	2.0 (1–9) (60)	-
	1–2	33.3 (45, 11.7)	1.5 (0.8; 3.0)	3.0 (2–11) (13)	0.8 (0.3–2.7)
	3–9	44.0 (25, 6.5)	2.4 (1.0; 5.5)	1.5 (1–4) (10)	3.2 (0.7–14.1)
	≥10	27.8 (18, 4.7)	1.2 (0.4; 3.4)	12.0 (9–20) (5)	3.0 (0.5–17.0)

^a^ domestic infestation was determined by the finding of at least one live bug by any of the bug collection methods used (i.e., timed-manual searches, during insecticide spraying operations, and householders’ bug collections).

^b^ bug abundance was calculated as the number of live bugs collected per 15 min-person among houses positive by timed-manual searches.

^c^ nesting indoors.

†includes all inhabited houses (n = 386).

OR: Crude odds ratio. RA: Relative abundance. CI: 95% confidence interval. Households with missing data were excluded for each variable. Age of house and domestic area were recorded in 2012.

*: CI not including 1.0.

**Table 3:** Distribution of domestic infestation prevalence and abundance of *T. infestans* according to sociodemographic variables, Pampa del Indio, Chaco, October 2008.

Variables		Infestation prevalence^a^ (no. of inspected houses, % of total)	OR (CI)†	Median bug abundance^b^ (first-third quartiles) (no. of infested houses)	RA (CI)†
Ethnic group					
	Qom	29.2 (346, 89.6)	3.7 (1.3; 10.7)*	2.0 (1–11) (85)	3.0 (0.9–10.6)
	Creole	10.0 (40, 10.4)	1	3.0 (1–28) (3)	-
Overcrowding^c^					
	< 1.0	0.0 (6, 2.2)	-	-	-
	1.0–2.0	17.2 (99, 35.9)	1	2.0 (1–3) (13)	-
	2.1–3.0	10.7 (56, 20.3)	0.6 (0.2; 1.6)	15 (5–28) (4)	1.7 (0.5–5.7)
	3.1–5.0	40.9 (66, 23.9)	3.3 (1.6; 6.8) *	2.0 (1–6) (24)	3.8 (1.2–10.9) *
	>5.0	42.9 (49, 17.8)	3.6 (1.7; 7.8) *	4.0 (1–21) (18)	7.0 (2.1–23.6) *
Household educational level^d^					
	< 6	30.1 (176, 59.5)	1	3.0 (1–11) (46)	-
	6–10	19.0 (100, 33.8)	0.5 (0.3; 1.0)	3.5 (1–12) (14)	0.4 (0.2–0.9) *
	11–13	17.7 (17, 5.7)	0.5 (0.1; 1.8)	1.5 (1–2) (2)	0.1 (0.01–0.6) *
	>13	0.0 (3, 1.0)	-	-	-
Insecticide use					
	Yes	21.2 (184, 47.7)	1	1.0 (1–11) (30)	-
	No	32.7 (202, 52.3)	1.8 (1.1; 2.9) *	3.0 (1–12) (58)	2.18 (1.04–4.57) *
Time since last insecticide spraying (years)					
	<2	23.1 (65, 21.5)	1	2.0 (1–4) (12)	-
	2–10	37.5 (8, 2.6)	2.0 (0.4; 9.4)	1.0 (-) (1)	0.1 (0.01–3.5)
	>10	33.3 (24, 7.9)	1.7 (0.6; 4.7)	3.0 (1–3) (5)	5.0 (0.9–27.6)
	Never	26.2 (206, 68.0)	1.2 (0.6; 2.3)	2.0 (1–12) (49)	2.2 (0.8–6.3)
Goat-equivalent index^e^					
	0	25.1 (179, 46.4)	1	2.0 (1–8) (39)	-
	1–5	33.6 (80, 20.7)	1.5 (0.8; 2.7)	4.0 (2–13) (25)	0.2 (0.03–1.4)
	6–30	40.4 (57, 14.8)	2.0 (1.1; 3.8) *	2.0 (1–9) (19)	0.5 (0.05–5.6)
	31–100	14.0 (43, 11.1)	0.5 (0.2; 1.2)	3.0 (2–58) (3)	0.1 (0.01–0.7) *
	>100	14.8 (27, 7.0)	0.5 (0.2; 1.6)	1.5 (1–2) (2)	-

^a^ domestic infestation was determined by the finding of at least one live bug by any of the bug collection methods used (i.e., timed-manual searches, during insecticide spraying operations, and householders’ bug collections).

^b^ bug abundance was calculated as the number of live bugs collected per 15 min-person among houses positive by timed-manual searches.

^c^ number of residents per sleeping quarter.

^d^ mean number of schooling years of household residents aged >15 years old.

^e^ total number of livestock (cows, pigs, goats) and poultry the household owns in terms of goat biomass.

† includes all inhabited houses (n = 386).

OR: crude odds ratio. RA: Relative abundance. CI: 95% confidence interval. Households with missing data were excluded for each variable. Number of rooms and household educational level were recorded in 2012.

*: CI not including 1.0.

Most Creole households applied insecticides in domestic premises (90.0%) and had window screens (59.5%), unlike Qom households (Table 1, S1 Text). Creole households applied high-concentration pyrethroid or carbamate insecticides (27.5%) much more frequently than Qom households (5.8%). Householders’ reports indicated that 68.0% of houses had never been sprayed with insecticides by vector control personnel whereas 21.5% had been sprayed two years before (Table 3). Local health personnel reportedly sprayed with insecticides 36 and 49 houses, mainly from Cuarta Legua villages in 2000 and 2006, respectively.

A key feature of the study population was the very frequent mobility of households to a new house (i.e., housing instability). Of all the inhabited houses enumerated in 2008, 20.2% (78) were demolished or abandoned by 2012, whereas 142 new houses were built over the four-year period, of which only 52 (36.7%) were present in 2012. Most of the new houses (90.1%) and the demolished or abandoned ones (98.7%) belonged to Qom households. Among the latter, movers were relatively disadvantaged compared to nonmovers. On average, movers had a lower goat-equivalent index (median, 0.5 versus 1.9); smaller domiciles (24 versus 36 m^2^); more recently-built houses (69.2% versus 45.6%); smaller household size (5.2 versus 6.8); and fewer peridomestic sites (1.3 versus 2.1).

### House infestation

*Triatoma infestans* was found by timed-manual searches in 108 (28.0%) of the 386 inhabited houses and in 6.9% of the 1,744 sites inspected. The median relative abundance was 3 bugs (first-third quartiles, 1–11) per unit of catch effort. Fifth-instar nymphs (24%), males (20%) and females (16%) were the stages most frequently captured. When the finding of bugs by any collection method was considered, the prevalence of house infestation slightly rose to 31.9% (123 of 386), and was 27.2% (105 of 386) in domestic sites and 7.8% (30 of 386) in peridomiciles. A total of 2,362 *T*. *infestans* was caught. *Triatoma sordida* was found in 4.5% (17 of 386) of houses exclusively in peridomiciles.

The contribution of each collection method to detection of domestic infestation is shown in S3 Table. Although the majority of domestic infestations was detected by timed-manual searches, bugs collected by householders (almost exclusively in domestic areas) and during insecticide spraying operations contributed to additional detection of 19 infested houses that timed searches had missed. Detection of domestic infestations by timed-manual searches and householders’ notifications were in poor agreement (kappa index = 0.3).

**Fig 2:**
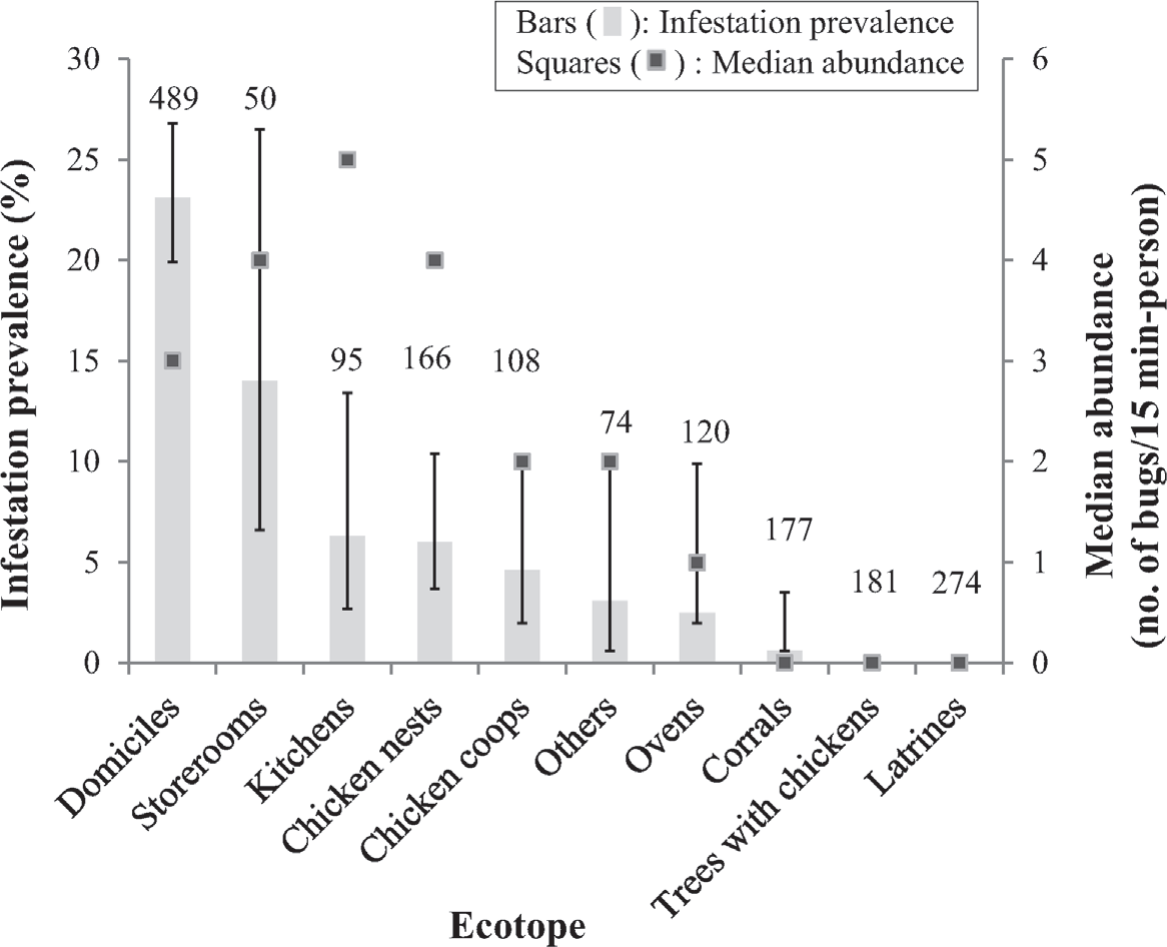
Prevalence of site-specific infestation (bars) and median abundance (squares) of *T*. *infestans* according to bug collection ecotope. Pampa del Indio, Chaco, October 2008. Infestation was determined by any bug collection method. Median bug abundance was calculated for houses found to be infested as determined by timed-manual collections. Whiskers indicate the upper and lower limits of the 95% CI for infestation prevalence. Numbers above bars indicate the number of sites inspected for infestation by timed-manual collections. “Others” mainly included small chapels, abandoned domiciles or vehicles, and stacked materials.

The ecotopes most frequently infested at site level (as determined by any bug collection method) were domiciles (23.1%), storerooms (14.0%), kitchens (6.3%), chicken nests (6.0%), and chicken coops (4.6%) (Fig. 2). The median abundance of *T*. *infestans* per unit of catch effort was higher in kitchens, storerooms and chicken nests, but did not differ significantly among ecotopes by negative binomial regression (P > 0.1 in all cases).

The overall prevalence of bug infection with *T*. *cruzi* was 25.0% among 719 live bugs examined, and ranged from 23.9% (150/628) in domiciles to 33.0% (30/91) in peridomiciles. Infected bugs were collected in domiciles of 45.2% (28/62) of the houses with bugs examined for infection, and in peridomiciles of 44.4% (8/18) of them.

**Fig 3:**
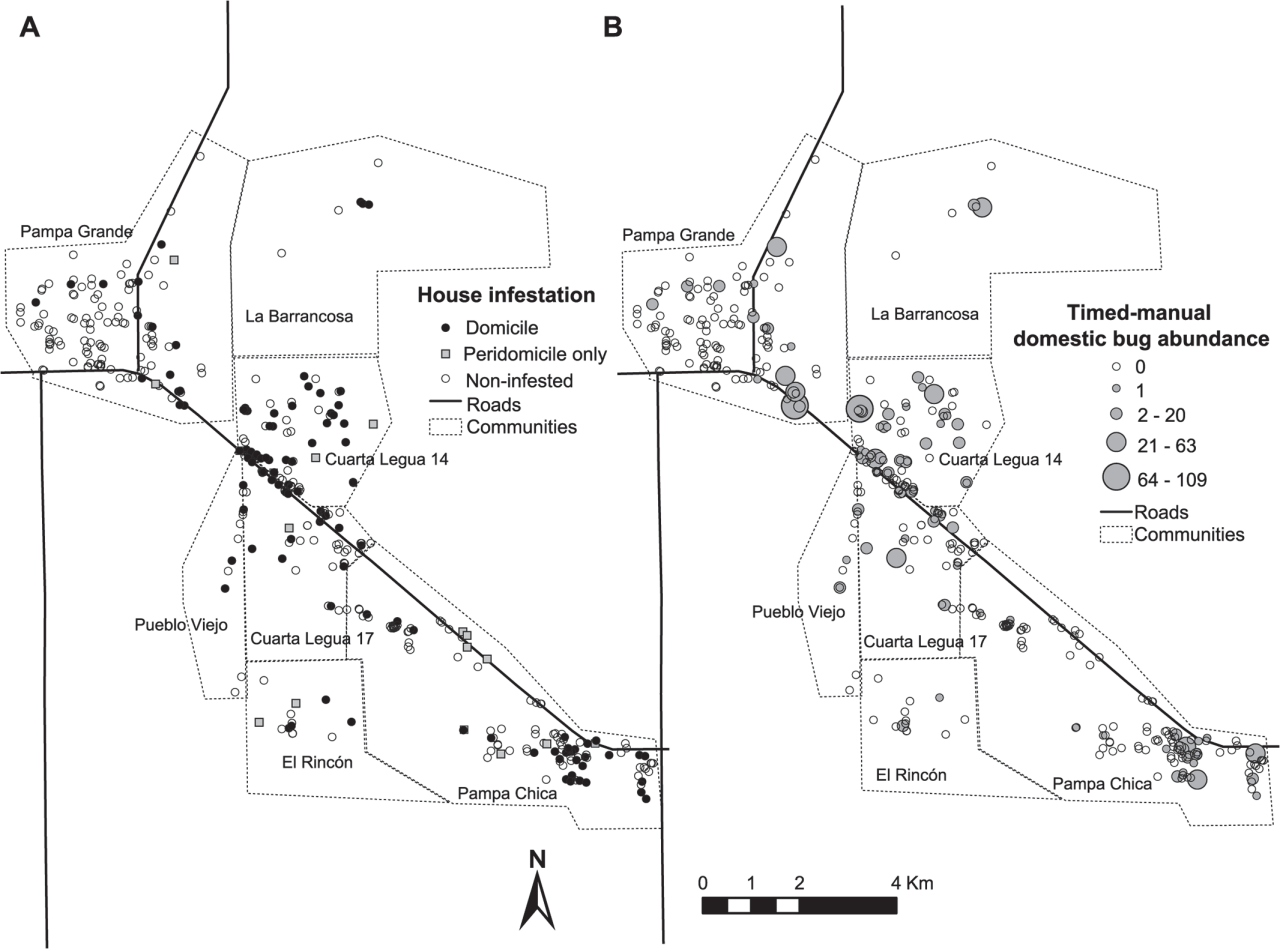
Spatial distribution of house infestation by *T*. *infestans*. Pampa del Indio, Chaco, October 2008. A. Distribution of domestic and peridomestic infestation as determined by any bug collection method. B. Distribution of domestic abundance according to timed-manual collections.

Although house infestation occurred across the study area, some communities showed larger domestic infestation than others (range, 12.2–50.8%) (Fig. 3, S1 Table). Domestic infestation was significantly aggregated at a global scale at distances ranging from 600 to 2,000 m (S2 Fig.); this means that infested houses were clustered, and on average, for every infested house there was a higher probability of finding another infested house within 600–2,000 m than expected by chance. Local spatial analyses of bug abundance identified clusters of houses located within 40–600 m in some communities (Cuarta Legua, Pampa Chica). The apparent cold spot at the NE angle (Pampa Grande village) was not statistically significant (P > 0.05).

### Domestic infestation, housing characteristics and sociodemographic variables

Wall and roof materials were significantly associated with domestic infestation (Table 2). The prevalence of domestic infestation increased significantly with increasing refuge availability levels and numbers of human residents, and declined steadily with increasing age of house and domestic area. Infested domiciles had a significantly smaller area (37.8 ± 27.2 m^2^, n = 84) than non-infested ones (50.6 ± 39.9 m^2^, n = 200; Mann-Whitney test, P < 0.001). Domestic infestation was higher in houses with at least one infested peridomestic site and fewer peridomestic structures (S1 Table). Domestic bug abundance only was significantly associated with refuge availability, domestic area and the number of dogs.

Qom households had a nearly threefold domestic infestation (29.2%) than Creoles’ (10.0%), whereas domestic bug abundance was similar between ethnic groups (Table 3). Domestic infestation increased steadily with increasing residential overcrowding from 0% up to 42.9%, and decreased with increasing household educational level from 30.1% to 0%. Households reporting insecticide use had a significantly lower infestation (21.2%) than those that did not (32.7%). Bug abundance was also significantly associated with residential overcrowding, household educational level, the goat-equivalent index, number of peridomestic sites, land ownership, and access to electricity (Table 3, S1 Table). The signs of the individual effects were the same as for domestic infestation. Households with no window screens had increased domestic infestation and bug abundance, whereas the use of bed nets was inversely associated (S1 Table). No significant association was found between domestic infestation and time since last insecticide spraying (Table 3).

### Multimodel inference

Using the first global model including 386 houses, we identified 11 and 6 models with considerable support (ΔAIC_c_ < 2) for domestic infestation and bug abundance, respectively. Refuge availability (RI = 1.00), distance to the nearest infested house (RI = 1.00–0.83) and insecticide use (RI = 0.75–0.69) were the most important factors (Table 4). Refuge availability exhibited a strong positive association whereas insecticide use and distance to the nearest infested house had a negative one. The presence of poultry indoors (RI = 0.75) was only moderately and directly associated with domestic bug abundance. The number of people (RI = 0.73) had moderate importance and marginally positive effects on infestation only, with CIs including the null value. Other factors presented lower RI for both response variables. Ethnicity showed low RI and widely variable CIs. The average logistic model for infestation (Hosmer-Lemeshow test, χ^2^ = 8.03; d.f. = 8; P = 0.43) and the area under the ROC curve (0.73) indicated a good fit. The model had moderate specificity (0.62) and sensitivity (0.71).

**Table 4:** Relative importance (RI), odds ratio (OR) and relative abundance (RA) for each variable regarding domestic infestation (logistic regression) and abundance of *T. infestans* (negative binomial regression) of all houses in the area (n = 386) and a subset with complete data for 12 variables (n = 274).

		Complete data set				Subset			
		Infestation^a^		Abundance^b^		Infestation^a^		Abundance^b^	
Variable		RI	OR (IC)	RI	RA (CI)	RI	OR (IC)	RI	RA (CI)
Refuge availability		**1**		**1**		**1**		**1**	
	3		1		1		1		1
	4		2.10 (1.09; 4.07)		8.94 (3.56–22.46)		2.08 (0.89; 4.85)		3.43 (1.19–9.87)
	5		4.38 (2.20; 8.71)		22.45 (8.43–59.80)		5.36 (2.27; 12.63)		20.39 (6.80–61.15)
Distance to nearest infested house^c^		**1**	0.92 (0.88; 0.97)	**0.83**	0.92 (0.86–0.98)	**0.88**	0.93 (0.87; 0.99)	**0.78**	0.90 (0.83–0.98)
Insecticide use		**0.75**		**0.69**		0.46		0.49	
	Yes		0.58 (0.34; 0.98)		0.46 (0.22–0.97)		0.64 (0.33; 1.24)		0.50 (0.20–1.23)
	No		1		1		1		1
No. of people		**0.73**	1.07 (1.00; 1.15)	0.44	1.08 (0.97–1.19)	0.53	1.07 (0.98; 1.18)	0.53	1.09 (0.97–1.23)
Presence of poultry^d^		0.64		**0.75**		0.53		0.56	
	Yes		1.70 (0.96; 2.96)		2.55 (1.08–6.01)		1.75 (0.86; 3.54)		2.21 (0.88–5.58)
	No		1		1		1		1
Infested peridomicile		0.65		0.30		0.51		0.28	
	Yes		2.38 (0.94; 6.05)		0.65 (0.15–2.83)		2.18 (0.78; 6.07)		0.73 (0.17–3.16)
	No		1		1		1		1
Ethnic group		0.52		0.29		0.30		0.35	
	Qom		2.50 (0.67; 9.25)		1.37 (0.27–7.02)		1.47 (0.38; 5.60)		0.50 (0.10–2.65)
	Creole		1		1		1		1
Goat-equivalent index^e^		0.35	1.02 (0.98; 1.05)	0.66	0.90 (0.80–1.01)	0.32	1.02 (0.98; 1.06)	0.58	0.89 (0.76–1.04)
No. of dogs or cats^d^		0.33	1.04 (0.94; 1.15)	0.46	1.12 (0.97–1.31)	0.40	1.07 (0.95; 1.21)	0.34	1.08 (0.93–1.26)
Housing quality		0.19		0.12		0.11		0.15	
	Low		1		1		1		1
	Intermediate		0.78 (0.44; 1.39)		0.85 (0.37–1.95)		1.04 (0.50; 2.17)		1.39 (0.50–3.92)
	High		0.67 (0.30; 1.51)		1.14 (0.38–3.39)		0.92 (0.34; 2.47)		1.64 (0.48–5.66)
Overcrowding^f^		-	-	-	-	**0.98**	1.25 (1.09; 1.44)	**1**	1.40 (1.18–1.65)
Educational level^g^		-	-	-	-	**0.74**	0.49 (0.25; 0.98)	0.68	0.45 (0.19–1.07)

^a^ domestic infestation was determined by the finding of at least one live bug by any of the bug collection methods used (i.e., timed-manual searches, during insecticide spraying operations, and householders’ bug collections).

^b^ bug abundance was calculated as the number of live bugs collected per 15 min-person among houses positive by timed-manual searches.

^c^ rescaled such that 1 unit is equal to 50 meters.

^d^ sleeping or nesting indoors.

^e^ total number of livestock (cows, pigs, goats, poultry) the household owns in terms of goat biomass. Rescaled such that 1 unit is equal to 10 goat-equivalents.

^f^. number of residents per sleeping quarter.

^g^ mean number of schooling years of household members aged >15 years old. Rescaled such that 1 unit is equal to 6 years of schooling.

Variables with high and moderate RI are shown in bold. The number of sleeping quarters and educational level were recorded in 2012.

For the second global model including 274 houses, refuge availability (RI = 1.00), overcrowding (RI > 0.98) and distance to the nearest infested house (RI = 0.78–0.88) were the most important factors and showed strong to moderate effects, whereas household educational level had moderate importance (RI = 0.74–0.68) and rather small negative effects on domestic infestation only (Table 4). The average logistic model for infestation (Hosmer-Lemeshow test χ^2^ = 3.66; d.f. = 8; P = 0.89) and the area under the ROC curve (0.79) indicated a good fit. This model showed higher specificity (0.71) and similar sensitivity (0.72). Removing overcrowding and household educational level data yielded results that were qualitatively similar to those in the first global model (not shown).

Additional analyses including only Qom households identified the same set of factors with high and moderate RI in both global models (not shown).

## Discussion

Our study identified important risk factors for domestic infestation and bug abundance in a rural area inhabited mainly by Qom populations through a multimodel inference framework. Some of these factors were novel and pertain to the sociodemographic domain, such as distance to the nearest infested house, residential overcrowding and household educational level, and accorded with predictions. We also corroborated the high RI of refuge availability and lack of domestic use of insecticides on both response variables, and the moderate RI of the number of people on domestic infestation recorded in the neighboring Area I [20]. These findings are in general qualitative agreement with the outcomes of studies on various species of Triatominae regardless of large differences between rural settings, ethnic composition, analytic methods and variables examined [20,25,27–32,37]. Surprisingly, the baseline prevalence of domestic infestation was much lower than expected on the basis of the near absence of insecticide spraying campaigns over the previous decade. This unexpected finding called for a post hoc explanation.

Domestic infestation and bug abundance increased with proximity to the nearest infested house although the effect was rather small. These observations are in agreement with predictions from patterns showing spatial aggregation of house infestation before and after insecticide spraying elsewhere in Argentina [20,56,57] but not in peri-urban habitats of Arequipa, Peru [58]. Subsequent studies in Arequipa, however, revealed that most of the infestations detected after the insecticide spraying campaign occurred in untreated houses which later served as sources of the insects that recolonized their neighbors [59]. The short distances between houses in several of our study villages clearly facilitated the invasion of *T*. *infestans* by flight or walking dispersal [60–62]. Both domestic infestation and bug abundance were spatially aggregated, with hotspots including heavily infested domiciles within 40–600 m; the upper bound is well within the observed flight dispersal range of *T*. *infestans* in a mark-recapture experiment [63]. Estimates based on the duration of sustained tethered flights suggest the flight range of *T*. *infestans* might exceed 2,400 m [64].

Residential overcrowding, a surrogate index for socioeconomic status and health conditions [65], was closely and positively associated with domestic infestation and bug abundance. This index approximates human density in sleeping quarters, and incorporates both household size and number of rooms in the domicile. Overcrowding is also expected to facilitate host finding and the human-feeding success of *T*. *infestans*, and most likely underlies the positive relation between the number of human occupants and domestic infestation by different species of triatomine bugs [24,28,58,66]. Likewise, the household seropositivity to *T*. *cruzi* increased steadily and significantly with decreasing size of domestic area –a putative index of stable settlement and well-being in Creole communities of the dry Chaco [67]. In our study, the average prevalence of domestic infestation also showed a strong positive trend with decreasing domestic area and age of house, and increasing number of people, although all CIs included the null values.

Household educational level averaged less than 6 years of schooling and was moderately important for domestic infestation, with negative effects whose size was rather small. Household education is considered a generic measure of household socioeconomic status, which is among the main social determinants of health inequalities [65]. The causal pathway linking increasing household educational levels to decreasing domestic infestation may be associated with access to information and receptivity to health education messages, which may translate into healthier practices [65]. For example, lower education levels correlated with increasing severity of Chagas disease cardiomyopathy, among other factors [68]. Even though education does not exclusively occur through formal instruction and duration of schooling does not specify its quality, educational level is a simple metric for comparison between households. Another measure of household socioeconomic status and wealth, the goat-equivalent index, showed a rather moderate RI for domestic bug abundance and marginally negative effects. The goat-equivalent index differed largely between ethnic groups (69×) and between movers and nonmovers; nearly one in every four Qom households owned no poultry and most had no livestock –stark measures of reduced livelihoods in a context where employment was rare and hunter-gatherer habits are no longer productive or feasible.

The application of domestic insecticides by householders was a moderately important factor negatively associated with domestic infestation and bug abundance as in other surveys [20,25,30], despite the fact that low-concentration sprays were mainly used against mosquitoes (S1 Text), as in other settings [69], and probably had very limited effects on triatomine bugs. Domestic insecticide use may be a surrogate for householders’ economic and behavioral aspects; its use implies both the capacity to purchase insecticides and willingness to take protective actions in response to nuisance insects. Two other protective practices (window screens and bed nets) showed opposite associations with ethnicity and domestic infestation in bivariate analyses. Window screens were restricted to brick-cement, Creole houses, and their presence was negatively associated with domestic bug infestation, as expected [70]. Conversely, only Qom households used bed nets, and their use was significantly and positively related to domestic infestation. This may be an example of reverse-causality effects which frequently limit the interpretation of associations derived from cross-sectional surveys. How well window screens and bed nets acted against triatomine bugs in the study setting remains to be determined.

House infestation with *T*. *infestans* (31.9%) was much lower than expected despite the fact that most houses provided suitable conditions for triatomines and the lack of insecticide spraying campaigns over the previous decade. Under similar circumstances, up to 90% of houses were infested elsewhere in the Gran Chaco [6,25,71]. This suggested that other additional factors may have affected the process of house reinfestation. Among the possible candidates we included: housing instability combined with a large fraction of recently-built houses; partial housing improvements (as shown by metal roofs); selective insecticide sprays; fewer peridomestic sites and fewer peridomestic foci of *T*. *infestans* than in other resource-constrained rural areas including Area I [20,57]. Indeed, the absence of a strong positive relationship between the occurrence of peridomestic foci and domestic infestation or bug abundance contradicts other findings elsewhere in the dry Chaco [7,56,57,72] and with various species of Triatominae [73,74], and may be explained by the paucity of peridomestic sites and domestic animals in Area III.

Housing instability was evidenced by the large mobility of Qom households within the study area and the municipality, and agreed with the large fraction of houses having less than 5 years of age at baseline. Residential mobility was virtually restricted to Qom households, indicating distinct patterns of settlement, housing occupation and displacement over time related to ethnicity. The backdrop for these patterns is the high population growth rate of local Qom population during recent decades, as evidenced by the very young age structure and decennial census figures, combined with intense out-migration to cities [19], in a context of structural poverty. Over 95% of the study population was native to the municipality of Pampa del Indio. The approximate stability of total population numbers in the study area is explained by out-migration with different destinations (S1 Text). Moreover, the highly skewed sex ratio toward males may be explained by gender-biased migration rates and accelerated population growth, but other competing hypotheses should be further investigated. The nature and implications of this rural-to-rural (local) movement are essentially different from unidirectional, rural-to-peri-urban migrations affecting house infestation elsewhere [75,76], and pose special challenges for research and vector control (see below). Housing instability, household mobility and migration patterns are key to understanding the process of house (re)infestation and to designing locally adapted vector surveillance systems in the Gran Chaco region.

Selective insecticide sprays performed by the local health system two years before our baseline survey were expected to lower house infestations, but a closer look revealed that the sprayed villages ranked at the top of the baseline infestation list (32.8–50.8%). The limited village-level effects of these treatments may in part be attributed to incomplete house coverage (37.1%); the occurrence of moderate resistance to pyrethroids in local *T*. *infestans* populations [39]; and fast reinfestation from untreated, neighboring sources (e.g.,[57,59]). Unfortunately, the selection criteria used to decide which villages and houses were sprayed could not be recalled by the participants in charge. If the sprayed villages had been selected based on having high infestation prevalence then we would expect them to have higher infestation rates than other villages two years later (as observed), given the partial spray coverage and strong association between preintervention and postintervention house infestation rates recorded in different settings (e.g.,[6,59,72,77]). Although householders reported additional insecticide treatments not registered by the local or provincial health system, 68.0% of responding households reported their current premises had never been treated with insecticides. Such low rates of spray coverage are roughly consistent with the average age of houses; the high rates of house destruction and reconstruction, and the dates and partial coverage of previous insecticide spraying campaigns.

Qom households had a threefold prevalence of domestic infestation than Creoles’, but the multivariate analyses revealed a large degree of uncertainty around the adjusted OR. The differential infestation between ethnic groups was greater than in Area I (34% versus 20%, respectively, Fig. 3 in [20]), where the Qom minority had similar domestic infestation levels as in Area III. Both areas and ethnic groups differed substantially in the mean number of poultry, livestock and other attributes (S2 Table compared with Table 2 in [20]), indicating that their living conditions were heterogeneous in several respects (inter-house distance, domestic area, household educational level, and domestic infestation, Table 1). Both in Area I and III, ethnicity had low RI when other important variables with a priori support were included in the global models, and the sizable differences in domestic infestation between ethnic groups presented a large degree of uncertainty. Other unidentified factors may underlie these heterogeneities within ethnic groups. Qom households likely were more infested because of the convergence of multiple factors intimately related to structural rural poverty (e.g., poor housing quality, overcrowding, less frequent insecticide use) rather than to direct ethnic or cultural effects facilitating house invasion and colonization by triatomine bugs. In fact, the increased mobility of Qom households most likely reduced house infestation and bug population size, and perhaps increased the spatial propagation of bugs. After accounting for the effects of other factors with high RI, ethnicity per se was a poor predictor of house infestation status.

Our study has some limitations and strengths. Because timed-limited manual searches underestimate the true house infestation rates at low levels of bug abundance [78], the additional bug collection methods were used to refine the assessment of infestation status. In general, householders’ bug collections (not bug notifications) have usually been more sensitive than timed-manual searches in domiciles across settings [8,78], but this pattern may vary with prior experience and promotion of vector surveillance activities (absent in our study area). Householders’ actual degree of compliance with bug collections usually remains unknown and is hard to gauge. Although some bias may result from the non-systematic bug collections performed by householders, searches conducted during insecticide spraying operations were systematic. Householders’ notification of domestic infestation by *T*. *infestans* had a poor agreement with timed-manual searches. This discrepancy is unlikely to be due to confusion with other insects because householders clearly distinguished *T*. *infestans* from other similar insects such as *T*. *sordida* (which showed low infestation prevalence in the area), and was perhaps related to notifying infestations that occurred in the past.

Selected sociodemographic variables measured four years after the baseline survey included a large number of missing data because the households that moved out were no longer reachable. Missing data frequently involve households with lower socioeconomic status [79], and in our study they apparently occurred at random (S1 Text). If household mobility was inversely associated with socioeconomic status (as suggested by the limited comparison between movers and nonmovers), the smaller sample with 274 houses could potentially be biased. However, the multimodel analysis of the global model reached similar qualitative conclusions regardless of the number of houses included, which suggests the magnitude of the bias was not serious. Variables based on householders’ reports of past events (e.g., insecticide sprays) may be affected by recall bias and social or cultural barriers against effective communication, including Spanish language skills. To minimize the latter, the questionnaires had previously been tested in other areas of the municipality and interviewees were acceptably fluent in Spanish. Some of the variables (e.g., land ownership, welfare support) may be open to social propriety issues and responses may have a variable degree of validity; for communal land ownership, however, responses matched other local sources of information. Information on the patterns of settlement, the extent of land owned by each household, income and employment may provide valuable, additional indicators of wealth and livelihood, but may also be affected by response bias. Major strengths of our study include the large number of Qom households surveyed over time and detailed household-level information on a sizable number of ecological and sociodemographic factors related to house infestation.

### Implications for vector and disease control

Our study documented threats of active vector-borne transmission of *T*. *cruzi* in approximately 27% of the households (as determined by the occurrence of domestic infestations), and identified manageable variables that may be targeted for improved interventions and risk stratification. Improving housing quality and living conditions is urgently needed and largely exceeds Chagas disease vector control because housing improvements will impact positively on family health. Reducing the presence of chickens in human sleeping quarters [20,21,25,26,64,67] and applying insecticides in more effective ways when required may contribute to improved vector control. Although these factors are frequently construed as environmental or ecological, the types of housing, land ownership, habits of raising livestock or poultry, frequency of insecticide use and type of preventive practices have historical, sociodemographic, cultural and political roots.

The household mobility patterns recorded have serious implications for vector and disease control. In the preintervention context of an infested area under marginal vector control (as in 2008), the mobility of Qom households implied the potential carriage of bugs in their belongings to the new houses, while leaving bugs behind in the rubble of knocked down walls. The recently-built houses represented new habitat patches susceptible to bug invasion and colonization, and therefore decreased the fraction of all houses effectively protected by the long-lasting residual effects of recent insecticide sprays. On the flip side, the processes of house destruction and reconstruction are expected to cause major negative impacts on the local abundance of bugs by increasing bug mortality and dispersal.

The mobility of some indigenous populations may pose special challenges to traditional housing improvement programs relying on stable settlement and secure land tenure. More knowledge of the drivers of household mobility, migration and the desired types of housing of Qom and other indigenous peoples which had a nomadic or seminomadic tradition are needed. The design of Chagas disease prevention programs and other health interventions directed to indigenous populations should address their specific needs and beliefs [80,81]. Improving housing quality in isolation, while traditional agricultural activities continue in decline and other sources of local employment are rare, may not stop the rural-to-peri-urban exodus across ethnic groups.

The links between household educational levels and domestic infestation require more elaboration and specific research on the mechanisms involved. This area offers new opportunities for innovative interventions through health education and promotion workshops [82] that include, but are not restricted to, community-based vector and disease surveillance, control and treatment. Better access to formal education may also contribute directly and indirectly to primary and secondary disease prevention (e.g., by increasing awareness of treatment opportunities). The large fraction of Qom and Creole households who managed to keep their premises free from triatomine bugs using the scarce means available to them holds promise for further improvements with a modest investment of resources. Households performing good practices of vector control may contribute as agents of change to further reduce infestation and transmission risks in community-based control programs.

The strong heterogeneities in the distribution of ecological and sociodemographic factors associated with house infestation may be used for risk stratification and targeted interventions. Large households residing in small-sized, precarious houses, with few or no livestock or poultry and lower educational levels, appear to be especially vulnerable for Chagas and other infectious diseases. These households and the affected communities may benefit from targeted disease prevention activities channeled through a more vigorous, adequately staffed, primary healthcare system deployed in the affected rural areas.

## Supporting Information

S1 Checklist STROBE Checklist. (DOCX)

S1 Fig Typical houses in the study area. Pampa del Indio, Chaco, 2008. Above: Domicile with mud walls. Below: House compound with two domiciles, including a new house provided by a social housing program.
(EPS)

S2 Fig Spatial analysis of domestic infestation by *T*. *infestans*. Pampa del Indio, Chaco, 2008. Dotted lines show 95% envelope intervals.
(EPS)

S1 Table Distribution of domestic infestation prevalence and abundance of *T*. *infestans* according to ecological and sociodemographic variables. Pampa del Indio, Chaco, October 2008. OR: Crude odds ratio. RA: Relative abundance. CI: 95% confidence interval. Infestation was determined by any bug collection method, and bug abundance by the number of live insects collected per 15 min-person. Households with missing data were excluded for each variable. *: CI not including 1.0.
(DOCX)

S2 Table Domestic hosts of each type per inhabited house compound according to resident ethnic group. Pampa del Indio, Chaco, October 2008.
(DOCX)

S3 Table Comparison of domestic infestation by *Triatoma infestans* as determined by each alternative method relative to the standard timed-manual collections with a dislodging aerosol. Pampa del Indio, Chaco, 2008.
(DOCX)

S4 Table Individual data including house infestation, environmental and sociodemographic factors. Pampa del Indio, Chaco, 2008.
(XLS)

S1 Text Additional population characteristics of the study area. Pampa del Indio, Chaco, October 2008.
(DOCX)

S2 Text Comparison among the negative binomial regression model for bug abundance with two mixture models (zero-inflated negative binomial and zero-inflated Poisson regressions) and a zero-augmented negative binomial regression model (“hurdle”). Pampa del Indio, Chaco, October 2008.
(DOCX)

## References

[1] Hotez PJ. Ten global “hotspots” for the neglected tropical diseases. PLoS Negl Trop Dis. 2014; 8(5):e2496 10.1371/journal.pntd.0002496 24873825 PMC4038631

[2] WHO. Neglected Tropical Diseases: Innovative and intensified disease management. 2013 [cited 2014 May 8]. Available from: http://www.who.int/neglected_diseases/disease_management/en/

[3] Segura EL. El control de la enfermedad de Chagas en la República Argentina In: Silveira AC, editor. El control de la enfermedad de Chagas en los países del Cono Sur de América. Historia de una iniciativa internacional. 1991/2001. Uberaba: Facultad de Medicina, Pan American Health Organization; 2002 p. 45–97.

[4] Silveira AC. El control de la Enfermedad de Chagas en los países del Cono Sur de América. Historia de una iniciativa internacional. 1991/2001 In: Silveira AC, editor. El control de la Enfermedad de Chagas en los países del Cono Sur de América. Historia de una iniciativa internacional. 1991/2001. Uberaba: Facultad de Medicina, Pan American Health Organization; 2002 p. 15–42.

[5] Gürtler RE. Sustainability of vector control strategies in the Gran Chaco Region: current challenges and possible approaches. Mem Inst Oswaldo Cruz. 2009; 104:52–9. 19753458 10.1590/s0074-02762009000900009 PMC3072747

[6] Gürtler RE, Kitron U, Cecere MC, Segura EL, Cohen JE. Sustainable vector control and management of Chagas disease in the Gran Chaco, Argentina. Proc Natl Acad Sci U S A. 2007; 104(41):16194–9. 17913895 10.1073/pnas.0700863104 PMC2042184

[7] Gorla DE, Porcasi X, Hrellac H, Catalá SS. Spatial stratification of house infestation by *Triatoma infestans* in La Rioja, Argentina. Am J Trop Med Hyg. 2009; 80(3):405–9. 19270290

[8] Rojas de Arias A, Ferro EA, Ferreira ME, Simancas LC. Chagas disease vector control through different intervention modalities in endemic localities of Paraguay. Bull World Health Organ. 1999; 77(4):331–9. 10327712 PMC2557652

[9] Naumann M. Atlas del Gran Chaco Sudamericano. Buenos Aires, Argentina: Sociedad Alemana de Cooperación Técnica (GTZ). ErreGé & Asoc.; 2006.

[10] Montenegro RA, Stephens C. Indigenous health in Latin America and the Caribbean. Lancet. 2006; 367(9525):1859–69. 16753489 10.1016/S0140-6736(06)68808-9

[11] Hotez PJ. Aboriginal populations and their neglected tropical diseases. PLoS Negl Trop Dis. 2014; 8(1):e2286 10.1371/journal.pntd.0002286 24498442 PMC3907312

[12] Gracey M, King M. Indigenous health part 1: determinants and disease patterns. Lancet. 2009; 374(9683):65–75. 10.1016/S0140-6736(09)60914-4 19577695

[13] Basombrio M, Segovia A, Peralta Ramos M, Esteban E, Stumpf R, Jurgensen P, et al Endemic *Trypanosoma cruzi* infection in indian populations of the Gran Chaco territory of South America: performance of diagnostic assays and epidemiological features. Ann Trop Med Parasitol. 1999; 93(1):41–8. 10492670 10.1080/00034989958780

[14] Biancardi MA, Conca Moreno M, Torres N, Pepe C, Altcheh J, Freilij H. Seroprevalencia de la Enfermedad de Chagas en 17 parajes del “Monte Impenetrable” de la provincia del Chaco. Medicina (Buenos Aires). 2003; 63(2):125–9. 12793080

[15] Sosa-Estani S, Dri L, Touris C, Abalde S, Dell’Arciprete A, Braunstein J. Vectorial and congenital transmission of *Trypanosoma cruzi* in Las Lomitas, Formosa. Medicina (Buenos Aires). 2009; 69(4):424–30. 19770096

[16] Moretti E, Castro I, Franceschi C, Basso B. Chagas disease: serological and electrocardiographic studies in Wichi and Creole communities of Misión Nueva Pompeya, Chaco, Argentina. Mem Inst Oswaldo Cruz. 2010; 105(5):621–7. 20835607 10.1590/s0074-02762010000500004

[17] Rojas de Arias A, de Guillen I, Inchausti A, Samudio M, Schmeda Hirschmann G. Prevalence of Chagas disease in Ayoreo communities of the Paraguayan Chaco. Trop Med Parasitol. 1993; 44:285–8. 8134769

[18] Samuels AM, Clark EH, Galdos-Cardenas G, Wiegand RE, Ferrufino L, Menacho S, et al Epidemiology of and impact of insecticide spraying on Chagas disease in communities in the Bolivian Chaco. PLoS Negl Trop Dis. 2013; 7(8):e2358 10.1371/journal.pntd.0002358 23936581 PMC3731239

[19] Valeggia CR, Tola F. The Argentine Toba In: Ember CR, Ember M, editors. Encyclopedia of medical anthropology: health and illness in the world’s cultures. New York: Kluwer Academic/Plenum Publishers; 2003 p. 564–72.

[20] Gurevitz JM, Ceballos LA, Gaspe MS, Alvarado-Otegui JA, Enriquez GF, Kitron U, et al Factors affecting infestation by *Triatoma infestans* in a rural area of the humid Chaco in Argentina: a multi-model inference approach. PLoS Negl Trop Dis. 2011; 5(10):e1349 10.1371/journal.pntd.0001349 22028941 PMC3196485

[21] Cardinal MV, Orozco MM, Enriquez GF, Ceballos LA, Gaspe MS, Alvarado-Otegui JA, et al Heterogeneities in the ecoepidemiology of *Trypanosoma cruzi* infection in rural communities of the Argentinean Chaco. Am J Trop Med Hyg. 2014; 90(6):1063–73. 10.4269/ajtmh.13-0251 24732461 PMC4047730

[22] Sommerfeld J, Kroeger A. Eco-bio-social research on dengue in Asia: a multicountry study on ecosystem and community-based approaches for the control of dengue vectors in urban and peri-urban Asia. Pathog Glob Health. 2012; 106(8):428–35. 10.1179/2047773212Y.0000000055 23318234 PMC3541880

[23] Mott KE, Muniz TM, Lehman JS Jr., Hoff R, Morrow RH Jr., de Oliveira TS, et al House construction, triatomine distribution, and household distribution of seroreactivity to *Trypanosoma cruzi* in a rural community in northeast Brazil. Am J Trop Med Hyg. 1978; 27(6):1116–22. 103445 10.4269/ajtmh.1978.27.1116

[24] Marsden P, Virgens D, Magalhaes I, Tavares-Neto J, Ferreira R, Costa CH, et al Ecologia domestica do *Triatoma infestans* em Mambaí, Goiás, Brasil. Rev Inst Med Trop Sao Paulo. 1982; 24(6):364–73. 6763760

[25] Cecere MC, Gürtler RE, Chuit R, Cohen JE. Factors limiting the domestic density of *Triatoma infestans* in north-west Argentina: a longitudinal study. Bull World Heal Organ. 1998; 76(4):373–84. 9803588 PMC2305757

[26] López A, Crocco L, Morales G, Catalá SS. Feeding frequency and nutritional status of peridomestic populations of *Triatoma infestans* from Argentina. Acta Trop. 1999; 73:275–81. 10546845 10.1016/s0001-706x(99)00039-x

[27] Enger KS, Ordoñez R, Wilson ML, Ramsey JM. Evaluation of risk factors for rural infestation by *Triatoma pallidipennis* (Hemiptera: Triatominae), a Mexican vector of Chagas disease. J Med Entomol. 2004; 41(4):760–7. 15311472 10.1603/0022-2585-41.4.760

[28] Campbell-Lendrum DH, Angulo VM, Esteban L, Tarazona Z, Parra GJ, Restrepo M, et al House-level risk factors for triatomine infestation in Colombia. Int J Epidemiol. 2007; 36(4):866–72. 17698884 10.1093/ije/dym065

[29] Bustamante DM, Monroy C, Pineda S, Rodas A, Castro X, Ayala V, et al Risk factors for intradomiciliary infestation by the Chagas disease vector *Triatoma dimidiata* in Jutiapa, Guatemala. Cad Saude Publica. 2009; 25:S83–92. 19287870 10.1590/s0102-311x2009001300008

[30] Saunders M, Small A, Dedicoat M, Roberts L. The development and validation of a risk score for household infestation by *Triatoma infestans*, a Bolivian vector of Chagas disease. Trans R Soc Trop Med Hyg. 2012; 106(11):677–82. 10.1016/j.trstmh.2012.07.006 22975298

[31] Dumonteil E, Nouvellet P, Rosecrans K, Ramirez-Sierra MJ, Gamboa-León R, Cruz-Chan V, et al Eco-bio-social determinants for house infestation by non-domiciliated *Triatoma dimidiata* in the Yucatan Peninsula, Mexico. PLoS Negl Trop Dis. 2013; 7(9):e2466 10.1371/journal.pntd.0002466 24086790 PMC3784500

[32] De Andrade AL, Zicker F, de Oliveira RM, Da Silva IG, Silva SA, de Andrade SS, et al Evaluation of risk factors for house infestation by *Triatoma infestans* in Brazil. Am J Trop Med Hyg. 1995; 53(5):443–7. 7485701 10.4269/ajtmh.1995.53.443

[33] Briceño-León R. La casa enferma Caracas: Fondo Editorial Acta Científica Venezolana; 1990.

[34] Bar-Yam Y. General features of complex systems Encyclopedia of Life Support Systems. Oxford, U.K.: EOLSS UNESCO Publishers; 2002.

[35] Charron DF. Ecosystem approaches to health for a global sustainability agenda. Ecohealth. 2012; 9(3):256–66. 10.1007/s10393-012-0791-5 22961374

[36] Ventura-Garcia L, Roura M, Pell C, Posada E, Gascón J, Aldasoro E, et al Socio-cultural aspects of Chagas disease: a systematic review of qualitative research. PLoS Negl Trop Dis. 2013; 7(9):e2410 10.1371/journal.pntd.0002410 24069473 PMC3772024

[37] Bustamante DM, De Urioste-Stone SM, Juárez JG, Pennington PM. Ecological, social and biological risk factors for continued *Trypanosoma cruzi* transmission by *Triatoma dimidiata* in Guatemala. Lazzari CR, editor. PLoS One. 2014; 9(8):e104599 10.1371/journal.pone.0104599 25170955 PMC4149347

[38] U.S. Census Bureau. Current Population Survey (CPS)—Definitions. [cited 2014 Nov 2]. Available from: http://www.census.gov/cps/about/cpsdef.html

[39] Gurevitz J, Gaspe M, Enriquez G, Vassena C, Alvarado-Otegui J, Provecho Y, et al Unexpected failures to control Chagas disease vectors with pyrethroid spraying in northern Argentina. J Med Entomol. 2012; 49(6):1379–86. 23270166 10.1603/me11157 PMC3760256

[40] Faust KA. Marriage, divorce, and family groups In: Siegel J, Swanson D, editors. The methods and materials of demography. Elsevier; 2004 p. 191–210.

[41] Ministry of Agriculture. Anuario 2010. Ganados y carnes. 2010 [cited 2014 May 8]. p. 452. Available from: http://www.minagri.gob.ar/site/ganaderia/anuario/pdf2010/ANUARIO 2010 COMPLETO web.pdf

[42] CEPAL. Panorama social de América Latina Santiago de Chile: Publicación de las Naciones Unidas; 1994.

[43] Instituto Nacional de Estadísticas y Censos (INDEC). Censo nacional de población, hogares y viviendas. 2010 [cited 2014 Apr 11]. Available from: http://www.censo2010.indec.gov.ar/

[44] Quantum GIS Development Team. Quantum GIS Geographic Information System: Release 2.4. Open Source Geospatial Foundation Project; 2014.

[45] Brown LD, Cai TT, DasGupta A. Interval estimation for a binomial proportion. Stat Sci. 2001; 16(2):101–33.

[46] Stata Corp. Stata Statistical Software. Release 12.0. College Station: Stata Corporation; 2011.

[47] Burnham KP, Anderson DR. Model selection and multimodel inference: a practical information-theoretic approach Springer-Verlag; 2002.

[48] Whittingham MJ, Stephens PA, Bradbury RB, Freckleton RP. Why do we still use stepwise modelling in ecology and behaviour? J Anim Ecol. 2006; 75(5):1182–9. 16922854 10.1111/j.1365-2656.2006.01141.x

[49] Burnham KP, Anderson DR. Multimodel Inference: Understanding AIC and BIC in Model Selection. Sociol Methods Res. 2004; 33(2):261–304.

[50] R Development Core Team. R: A language and environment for statistical computing. Release 2.15.1 Vienna: R Foundation for Statistical Computing; 2012.

[51] Fortin MJ, Dale MRT. Spatial analysis: A guide for ecologists Cambridge University Press; 2005.

[52] Wiegand T, Moloney KA. Rings, circles, and null-models for point pattern analysis in ecology. 2004; 104:209–29.

[53] Getis A, Ord JK. Local spatial statistics: an overview In: Longley P, Batty M, editors. Spatial analysis modelling in a GIS environment. GeoInformation International; 1996 p. 269–85.

[54] Chen D, Getis A. Point Pattern Analysis (PPA). San Diego: San Diego State University; 1998.

[55] U. N. Population Division. Population trends. [cited 2014 Nov 4]. Available from: http://www.un.org/en/development/desa/population/theme/trends/index.shtml

[56] Cecere MC, Vazquez-Prokopec GM, Ceballos LA, Gurevitz JM, Zárate JE, Zaidenberg M, et al Comparative trial of effectiveness of pyrethroid insecticides against peridomestic populations of *Triatoma infestans* in northwestern Argentina. J Med Entomol. 2006; 43(5):902–9. 17017227 10.1603/0022-2585(2006)43[902:ctoeop]2.0.co;2 PMC1894891

[57] Cecere MC, Vazquez-Prokopec GM, Gürtler RE, Kitron U. Spatio-temporal analysis of reinfestation by *Triatoma infestans* (Hemiptera: Reduviidae) following insecticide spraying in a rural community in northwestern Argentina. Am J Trop Med Hyg. 2004; 71(6):803–10. 15642975 PMC1351234

[58] Levy MZ, Bowman NM, Kawai V, Waller LA, Cornejo del Carpio JG, Cordova Benzaquen E, et al Periurban *Trypanosoma cruzi*-infected *Triatoma infestans*, Arequipa, Peru. Emerg Infect Dis. 2006; 12(9):1345–52. 17073082 10.3201/eid1209.051662 PMC3294737

[59] Barbu C, Buttenheim A, Hancco Pumahuanca M, Quintanilla Calderón J, Salazar R, Niemierko M, et al Residual infestation and recolonization in an urban *Triatoma infestans* control campaign. Emerg Infect Dis. 2014; 20(12):2055–63. 10.3201/eid2012.131820 25423045 PMC4257819

[60] Vazquez-Prokopec GM, Ceballos LA, Marcet PL, Cecere MC, Cardinal MV, Kitron U, et al Seasonal variations in active dispersal of natural populations of *Triatoma infestans* in rural north-western Argentina. Med Vet Entomol. 2006; 20(3):273–9. 17044877 10.1111/j.1365-2915.2006.00637.x PMC1894892

[61] Abrahan LB, Gorla DE, Catalá SS. Dispersal of *Triatoma infestans* and other Triatominae species in the arid Chaco of Argentina: flying, walking or passive carriage? The importance of walking females. Mem Inst Oswaldo Cruz. 2011; 106(2):232–9. 21537686 10.1590/s0074-02762011000200019

[62] Barbu CM, Hong A, Manne JM, Small DS, Quintanilla Calderón JE, Sethuraman K, et al The effects of city streets on an urban disease vector. PLoS Comput Biol. 2013; 9(1):e1002801 10.1371/journal.pcbi.1002801 23341756 PMC3547802

[63] Schofield CJ, Lehane MJ, McEwen PK, Catalá SS, Gorla DE. Dispersive flight by *Triatoma infestans* under natural climatic conditions in Argentina. Med Vet Entomol. 1992; 6(1):51–6. 1600228 10.1111/j.1365-2915.1992.tb00035.x

[64] Gürtler RE, Cecere MC, Fernández MDP, Vazquez-Prokopec GM, Ceballos LA, Gurevitz JM, et al Key source habitats and potential dispersal of *Triatoma infestans* populations in northwestern Argentina: Implications for vector control. PLoS Negl Trop Dis. 2014; 8(10):e3238 10.1371/journal.pntd.0003238 25299653 PMC4191936

[65] WHO. A conceptual framework for action on the social determinants of health. Geneva, Switzerland; 2010 p. 75.

[66] Piesman J, Sherlock IA, Christensen HA. Host availability limits population density of *Panstrongylus megistus* . Am J Trop Med Hyg. 1983; 32(6):1445–50. 6359911 10.4269/ajtmh.1983.32.1445

[67] Gürtler RE, Chuit R, Cecere MC, Castañera MB, Cohen JE, Segura EL. Household prevalence of seropositivity for *Trypanosoma cruzi* in three rural villages in northwest Argentina: environmental, demographic, and entomologic associations. Am J Trop Med Hyg. 1998; 59(5):741–9. 9840591 10.4269/ajtmh.1998.59.741

[68] Viotti R, Vigliano CA, Álvarez MG, Lococo BE, Petti MA, Bertocchi GL, et al El impacto de las condiciones socioeconómicas en la evolución de la Enfermedad de Chagas crónica. Rev Española Cardiol. 2009; 62(11):1224–32. 19889333 10.1016/s1885-5857(09)73349-3

[69] Rosecrans K, Cruz-Martin G, King A, Dumonteil E. Opportunities for improved chagas disease vector control based on knowledge, attitudes and practices of communities in the yucatan peninsula, Mexico. PLoS Negl Trop Dis. 2014; 8(3):e2763 10.1371/journal.pntd.0002763 24676038 PMC3967964

[70] Ferral J, Chavez-Nuñez L, Euan-Garcia M, Ramirez-Sierra MJ, Najera-Vazquez MR, Dumonteil E. Comparative field trial of alternative vector control strategies for non-domiciliated *Triatoma dimidiata* . Am J Trop Med Hyg. 2010; 82(1):60–6. 10.4269/ajtmh.2010.09-0380 20064997 PMC2803511

[71] Paulone I, Chuit R, Perez AC, Canale DM, Segura EL. The status of transmission of *Trypanosoma cruzi* in an endemic area of Argentina prior to control attempts, 1985. Ann Trop Med Parasitol. 1991; 85(5):489–97. 1809241 10.1080/00034983.1991.11812598

[72] Cecere MC, Vazquez-Prokopec GM, Ceballos LA, Boragno S, Zárate JE, Kitron U, et al Improved chemical control of Chagas disease vectors in the Dry Chaco region. J Med Entomol. 2013; 50(2):394–403. 23540129 10.1603/me12109 PMC3773707

[73] Cohen JM, Wilson ML, Cruz-Celis A, Ordoñez R, Ramsey JM. Infestation by *Triatoma pallidipennis* (Hemiptera: Reduviidae: Triatominae) is associated with housing characteristics in rural Mexico. J Med Entomol. 2006; 43(6):1252–60. 17162961 10.1603/0022-2585(2006)43[1252:ibtphr]2.0.co;2

[74] Walter A, do Rego IP, Ferreira AJ, Rogier C. Risk factors for reinvasion of human dwellings by sylvatic triatomines in northern Bahia State, Brazil. Cad Saude Publica. 2005; 21(3):974–8. 15868058 10.1590/s0102-311x2005000300034

[75] Bayer AM, Hunter GC, Gilman RH, Cornejo del Carpio JG, Naquira-Velarde C, Bern C, et al Chagas disease, migration and community settlement patterns in Arequipa, Peru. PLoS Negl Trop Dis. 2009; 3(12):e567 10.1371/journal.pntd.0000567 20016830 PMC2790340

[76] Levy MZ, Barbu CM, Castillo-Neyra R, Quispe-Machaca VR, Ancca-Juarez J, Escalante-Mejia P, et al Urbanization, land tenure security and vector-borne Chagas disease. Proc Biol Sci. 2014; 281(1789):1–9. 10.1098/rspb.2014.1003 PMC4100517 24990681

[77] Gürtler RE, Canale DM, Spillmann C, Stariolo R, Salomon OD, Blanco S, et al Effectiveness of residual spraying of peridomestic ecotopes with deltamethrin and permethrin on *Triatoma infestans* in rural western Argentina: a district-wide randomized trial. Bull World Heal Organ. 2004; 82(3):196–205. 15112008 PMC2585938

[78] Abad-Franch F, Vega MC, Rolón MS, Santos WS, Rojas de Arias A. Community participation in Chagas disease vector surveillance: systematic review. PLoS Negl Trop Dis. 2011; 5(6):e1207 10.1371/journal.pntd.0001207 21713022 PMC3119642

[79] Cortinovis I, Vella V, Ndiku J. Construction of a socio-economic index to facilitate analysis of health data in developing countries. Soc Sci Med. 1993; 36(8):1087–97. 8475425 10.1016/0277-9536(93)90127-p

[80] Dell’Arciprete A, Braunstein J, Touris C, Dinardi G, Llovet I, Sosa-Estani S. Cultural barriers to effective communication between indigenous communities and health care providers in northern Argentina: an anthropological contribution to Chagas disease prevention and control. Int J Equity Health. 2014; 13(1):6. 24476151 10.1186/1475-9276-13-6 PMC3909457

[81] Dib J, Agudelo L, Vélez I. Prevalencia de patologías tropicales y factores de riesgo en la comunidad indígena de Bunkwimake, Sierra Nevada de Santa Marta. Rev Fac Ciencias Salud, Colombia. 2006; 3(1):38–44.

[82] Crocco L, Rodríguez C, Catalá SS, Nattero J. Chagas disease in Argentina: tools for schoolchildren to exercise vector surveillance and identify household risk factors. Cad Saude Publica. 2005; 21(2):646–51. 15905931 10.1590/s0102-311x2005000200034

