# Supplementary material for: Ecological and Sociodemographic Determinants of House Infestation by Triatoma infestans in Indigenous Communities of the Argentine Chaco
Source: PLoS Negl Trop Dis. 2015 Mar 18;9(3):e0003614. doi: 10.1371/journal.pntd.0003614 (PMC4364707; doi:10.1371/journal.pntd.0003614)
Supplement: S1 Table — Pampa del Indio, Chaco, October 2008. OR: Crude odds ratio. RA: Relative abundance. CI: 95% confidence interval. Infestation was determined by any bug collection method, and bug abundance by the number of live insects collected per 15 min-person. Households with missing data were excluded for each variable. *: CI not including 1.0. (DOCX) [file pntd.0003614.s004.docx]

**Table S1. Distribution of domestic infestation prevalence and abundance of *T. infestans* according to ecological and sociodemographic variables**. Pampa del Indio, Chaco, October 2008. OR: Crude odds ratio. RA: Relative abundance. CI: 95% confidence interval. Infestation was determined by any bug collection method, and bug abundance by the number of live insects collected per 15 min-person. Households with missing data were excluded for each variable. *: CI not including 1.0.

| Variable | Infestation prevalence (no. of inspected houses, % of total) | OR (CI)† | Median bug abundance (1st-3rd quartiles) (no. of infested houses) | RA (CI)† |
| --- | --- | --- | --- | --- |
| Community |  |  |  |  |
| Pampa Grande | 12.2 (115, 29.8) | 1 | 12.0 (2-21) (14) | - |
| Pampa Chica | 25.0 (120, 31.1) | 2.4 (1.2; 4.8)* | 1.0 (1-4) (23) | 0.6 (0.2-1.6) |
| Cuarta Legua 14 | 50.8 (63, 16.3) | 7.4 (3.5; 15.7)* | 2.0 (1-7) (31) | 1.7 (0.6-5.2) |
| Cuarta Legua 17 | 32.8 (58, 15.0) | 3.5 (1.6; 7.7)* | 3.0 (1-11) (13) | 0.6 (0.2-1.9) |
| La Barrancosa | 50.0 (6, 1.6) | 7.2 (1.3; 39.3)* | 2.0 (1-32) (3) | 2.4 (0.1-46.3) |
| Pueblo Viejo | 30.0 (10, 2.6) | 3.1 (0.7; 13.3) | 1.5 (1-2) (2) | 0.1 (0.01-1.6) |
| El Rincón | 28.6 (14, 3.6) | 2.9 (0.8; 10.4) | 6.5 (1-12) (2) | 0.4 (0.1-3.0) |
| No. of peridomestic sites |  |  |  |  |
| 0 | 30.0 (100, 25.9) | 1 | 5.0 (1-12) (27) | - |
| 1 | 30.1 (93, 24.1) | 1.0 (0.5; 1.9) | 2.0 (1-7) (25) | 0.5 (0.2-1.3) |
| 2-5 | 26.2 (145, 37.6) | 0.8 (0.5; 1.5) | 3.0 (1-10) (32) | 0.4 (0.2-1.0) |
| >5 | 18.8 (48, 12.4) | 0.4 (0.2; 1.2) | 1.0 (1-2) (4) | 0.02 (0.01-0.1)* |
| Infested peridomicile |  |  |  |  |
| Yes | 40.0 (30, 7.8) | 1.9 (0.9; 4.1) | 3.0 (1-9) (6) | 0.9 (0.2-3.8) |
| No | 26.1 (356, 92.2) | 1 | 2.0 (1-11) (82) | - |
| No. of dogs indoors^a^ |  |  |  |  |
| 0 | 24.3 (214, 55.4) | 1 | 2.0 (1-8) (42) | - |
| 1-2 | 24.6 (61, 15.8) | 1.0 (0.5; 2.0) | 2.5 (1-20) (14) | 3.5 (1.2-9.7)* |
| ≥3 | 34.2 (111, 28.8) | 1.6 (1.0; 2.7) | 3.0 (1-10) (32) | 2.0 (0.8-4.5) |
| No. of cats indoors ^a^ |  |  |  |  |
| 0 | 25.7 (257, 66.9) | 1 | 3.0 (1-12) (54) | - |
| 1-2 | 29.7 (111, 28.9) | 1.2 (0.8; 2.0) | 2.5 (1-9) (28) | 0.9 (0.4-2.2) |
| ≥3 | 37.5 (16, 4.2) | 1.7 (0.6; 5.0) | 1.5 (1-2) (6) | 0.3 (0.04-1.9) |
| Land ownership |  |  |  |  |
| Individual | 20.4 (113, 39.8) | 1 | 2.0 (1-8) (16) | - |
| Familial | 28.9 (104, 36.6) | 1.6 (0.9; 3.0) | 3.0 (1-11) (28) | 3.3 (1.3-8.8)* |
| Communal | 28.2 (39, 13.7) | 1.5 (0.7; 3.5) | 3.0 (1-12) (9) | 3.5 (0.9-13.1) |
| None | 25.0 (28, 9.9) | 1.3 (0.5; 3.4) | 2.0 (1-4) (6) | 0.9 (0.2-4.3) |
| Window screen |  |  |  |  |
| Yes | 18.0 (39, 14.4) | 1 | 1.0 (-) (5) | - |
| No | 26.8 (231, 85.6) | 1.7 (0.7; 4.0) | 3.0 (1-11) (53) | 5.2 (1.4-19.6)* |
| Bed net |  |  |  |  |
| Yes | 39.5 (81, 30.2) | 1 | 3.0 (1-11) (29) | - |
| No | 19.8 (187, 69.8) | 0.4 (0.2; 0.7)* | 2.0 (1-8) (29) | 0.2 (0.1-0.6)* |
| Agricultural activities |  |  |  |  |
| Yes | 23.0 (165, 84.6) | 1 | 1.0 (1-8) (33) | - |
| No | 23.3 (30, 15.4) | 1.0 (0.4; 2.6) | 1.0 (1-25) (6) | 1.4 (0.3-6.1) |
| Participation in social organizations |  |  |  |  |
| Yes | 25.0 (80, 31.5) | 1 | 3.0 (1-20) (15) | - |
| No | 24.1 (174, 68.5) | 1.0 (0.5; 1.8) | 3.0 (1-9) (36) | 0.7 (0.3-1.8) |
| Electricity |  |  |  |  |
| Yes | 18.6 (70, 27.0) | 1 | 3.0 (1-4) (11) | - |
| No | 28.6 (189, 73.0) | 1.8 (0.9; 3.5) | 3.0 (1-12) (43) | 3.8 (1.4-10.7)* |

^a^ sleeping indoors.

† includes all inhabited houses (n = 386).
