# Supplementary material for: Ecological and Sociodemographic Determinants of House Infestation by Triatoma infestans in Indigenous Communities of the Argentine Chaco
Source: PLoS Negl Trop Dis. 2015 Mar 18;9(3):e0003614. doi: 10.1371/journal.pntd.0003614 (PMC4364707; doi:10.1371/journal.pntd.0003614)
Supplement: S2 Table — Pampa del Indio, Chaco, October 2008. (DOCX) [file pntd.0003614.s005.docx]

| Mean no. per house ± standard deviation (% households with host) | | | |
| --- | --- | --- | --- |
| Host | Qom | Creole | Total |
| People | 6.4 ± 3.6 (100) | 4.1 ± 2.2 (100) | 6.2 ± 3.6 (100) |
| 0-5 yr old | 1.2 ± 1.2 (62.7) | 0.4 ± 0.7 (30) | 1.1 ± 1.1 (59.3) |
| 6-14 yr old | 1.8 ± 1.8 (67.9) | 1.0 ± 1.3 (50) | 1.7 ± 1.8 (66.1) |
| Poultry | 16.7 ± 21.5 (76.8) | 51.6 ± 48.7 (87.2) | 20.3 ± 27.6 (77.9) |
| Goats | 3.1 ± 8.8 (24.3) | 20.1 ± 32.0 (48.7) | 4.9 ± 14.1 (26.8) |
| Pigs | 1.3 ± 3.4 (21.4) | 4.7 ± 7.5 (56.4) | 1.6 ± 4.2 (25.0) |
| Cows | 1.4 ± 14.5 (5.6) | 25.9 ± 47.2 (59.0) | 3.9 ± 21.6 (11.1) |
| Equines | 0.7 ± 0.2 (17.9) | 8.4 ± 24.0 (66.7) | 1.5 ± 8.2 (22.9) |

**Table S2. Domestic hosts of each type per inhabited house compound according to resident ethnic group.** Pampa del Indio, Chaco, October 2008.
