# Supplementary material for: Ecological and Sociodemographic Determinants of House Infestation by Triatoma infestans in Indigenous Communities of the Argentine Chaco
Source: PLoS Negl Trop Dis. 2015 Mar 18;9(3):e0003614. doi: 10.1371/journal.pntd.0003614 (PMC4364707; doi:10.1371/journal.pntd.0003614)
Supplement: S3 Table — Pampa del Indio, Chaco, 2008. (DOCX) [file pntd.0003614.s006.docx]

Table S3. Comparison of domestic infestation by *Triatoma infestans* determined by each alternative method relative to the standard timed-manual collections using a dislodging aerosol. Pampa del Indio, Chaco, 2008.

| Alternative method | No. of houses inspected | N° positive houses: | | | N° negative by both methods |
| --- | --- | --- | --- | --- | --- |
|  |  | By both methods | Only by standard method | Only by alternative method |  |
| Householders’ bug collections | 386 | 20 | 68 | 10^a^ | 288 |
| During insecticide spraying | 386 | 19 | 69 | 10^a^ | 288 |
| Total^b^ | 386 | 41 | 47 | 19 | 279 |
| Householders’ bug notifications^c^ | 371 | 63 | 22 | 88 | 198 |

^a^ Includes two houses negative by timed searches which were only considered in this table and not in subsequent analyses because the bug collection site was not specified. One of the houses was found to be infestedduring insecticide spraying and by householders’ collections and the other one only by the latter method.
^b^ Infested by at least one method showing the occurrence of bugs (i.e., timed searches, bug collections during insecticide spraying and householders’ bug collections).

^c^ Excludes 15 houses with missing data; three of them were infested.
