# Supplementary material for: Ecological and Sociodemographic Determinants of House Infestation by Triatoma infestans in Indigenous Communities of the Argentine Chaco
Source: PLoS Negl Trop Dis. 2015 Mar 18;9(3):e0003614. doi: 10.1371/journal.pntd.0003614 (PMC4364707; doi:10.1371/journal.pntd.0003614)
Supplement: S1 Text — Pampa del Indio, Chaco, October 2008. (DOCX) [file pntd.0003614.s008.docx]

Text S1. **Additional population characteristics of the study area.** Pampa del Indio, Chaco, October 2008.

Georeferenced house locations

The geographical coordinates of each house compound were transformed to preserve the privacy of the households involved in this study. The points were jittered in their X and Y coordinates by adding random noise within 214 meters of each house. This value was 1.5 times the average distance among house compounds and represented 1.5% of the maximum distance in X and Y of the area. The coordinates also were shifted according to a new reference point which consisted of the minimum X and Y coordinates in the UTM coordinate system.

Missing data

To test for randomness of the missing data we used Pearson product-moment correlations as follows: if a variable had missing data, we constructed a dummy variable (zero was assigned to all cases in which the value was missing and one when it was recorded), and calculated pairwise correlation coefficients between the dummy variables and the 9 variables with complete data. All correlation coefficients were non-significant (r < 0.2), suggesting that the distribution of missing values was completely at random.

We assumed that the missing values were related to the frequent mobility of households. To verify it, we calculated correlation coefficients between variables with missing data and the new variable “housing instability” (with zero assigned to houses that existed in 2008 but not in 2012, and one to houses that existed in both surveys). The coefficients ranged from 0.49-0.63 for most variables, suggesting that the underlying reason for missing data was housing instability, except for “age of house” (r = 0.27) and “time since last insecticide spraying” (r = 0.04) in which missing data were completely at random. Therefore, since list-wise deletion was used in the multivariate analysis of the subset database and assuming that housing instability would affect house infestation status, the outcome of the subset analysis may be biased toward stable households.

House-level characteristics

An average household had 6.2 members, 20.3 chickens, 4.9 goats, 1.6 pigs, 3.9 cows and 1.5 equines, with large differences between ethnic groups (Table S2). More than 50% of households had 3 or more residents per sleeping quarter (i.e., critical overcrowding). The average size of domestic sites (46.8 m^2^; SD, 35.8) steadily increased with increasing age of the house, from 41.7 m^2^ in those aged less than one year, to 42.4 m^2^, 53.9 m^2^, 50.6 m^2^ and 69.4 m^2^ in houses with 2-5, 6-10, 11-20 and more than 20 years of age, respectively. Unlike in a contiguous rural section (Area I) mainly inhabited by Creoles [20], most houses had few peridomestic sites (2.4 ± 0.1), and a large fraction only had domestic sites (25.9%) and more than one domicile per house compound (19.2%). An average Qom household had 6.4 occupants in a 43.4-m^2^ domicile with only one room, whereas local Creoles had 4.1 occupants in a nearly twice as large area. The application of low-concentration pyrethroid sprays (mostly against mosquitoes) was reported by 55% of Creole and 31.2% of Qom households. According to householders’ reports, 71.2% of Qom and 54.3% of Creole households received public welfare support regularly.

Activities and land ownership

Only 27.0% of households reported having electricity (Table 1) and 52% had access to potable water, mostly from communal taps. Most households (>79%) had a subsistence economy based on agriculture for self-consumption. Agricultural activities reportedly declined over the previous years owing to recurrent draughts, the reduced market price of cotton, and lack of state support for agriculture. Most households reported individual or familial land ownership (>76%). Only Qom households reported communal land ownership (15.9%).

Mobility

Among Qom households that moved between 2008 and 2012 and could be relocated, 52% moved within the study area to a newly-built house or to a relative’s house; 22% moved to urban or peri-urban areas of Pampa del Indio town; 3.8% to distant cities; in 7.7% of houses the only resident passed away; and in 12% the new destination of the emigrating households could not be identified. When asked for the cause of local displacement, movers who could be relocated reported the search of better lands to cultivate; access to electricity, potable water, health care and education; conflict with neighbors; and leaving the house when the head of the household died (i.e., a Qom tradition).
