# Supplementary material for: Ecological and Sociodemographic Determinants of House Infestation by Triatoma infestans in Indigenous Communities of the Argentine Chaco
Source: PLoS Negl Trop Dis. 2015 Mar 18;9(3):e0003614. doi: 10.1371/journal.pntd.0003614 (PMC4364707; doi:10.1371/journal.pntd.0003614)
Supplement: S2 Text — Pampa del Indio, Chaco, October 2008. (DOCX) [file pntd.0003614.s009.docx]

Text S2. **Comparison among the negative binomial regression model for bug abundance with two mixture models (zero-inflated negative binomial and zero-inflated Poisson regressions) and a zero-augmented negative binomial regression model (“hurdle”).** Pampa del Indio, Chaco, October 2008.

Mixture and two-part models (zero-inflated models) can model excess of zeros when the response variable contains more zeros than expected from a negative binomial distribution. Although negative binomial regressions capture over-dispersion well, zero-inflated models add a second model component capturing zero counts (Zeileis et al. 2008). These models are used when over-dispersion is due to an excessive number of zeros rather than to variation in the count data. In our data set, excess of zeros in bug abundance could be due to the limited sensitivity of the bug collection method (false negatives) or to the absence of infestation (true negatives). In addition, over-dispersion in the count data may arise from the aggregated nature of house infestation by triatomine bugs. Therefore, we compared the negative binomial regression model (NB) with two mixture models: a zero-inflated negative binomial regression (ZINB) and a zero-inflated Poisson regression (ZIP); and a two-part model: a zero-augmented negative binomial regression (ZANB, also known as “hurdle”).

Mixture and two-part models differ in how they deal with the different types of zeros. The two-part models consist of two parts: a binomial model used to model the probability that a zero value is observed, and the second part consists of a zero-truncated Poisson or negative binomial model. Mixture models also include two different processes (i.e. the binomial process and the count process) but the count process (Poisson or negative binomial model) is not zero truncated (Zuur et al. 2009). Two-part models do not discriminate between false and true negatives, whereas mixture models can potentially differentiate between them if the covariates used explain the probability of the event (i.e., domestic infestation) thoroughly (Zuur et al. 2009).

We compared the models using the Vuong's non-nested hypothesis test, which is based on a comparison of the predicted probabilities of two models that are not nested, and allows the comparison between zero-inflated count models and their non-zero-inflated analogs (Vuong 1989).

We ran the zero-inflated models for bug abundance in R using the “pscl” package and compared them to the negative binomial model using the “MASS” package, both for the complete dataset and the subset data. We selected as covariates those variables that showed a significant effect (p < 0.05) in the zero part of the hurdle model. The same four covariates were included in all two-part/mixture models: presence of poultry indoors, use of insecticide, refuge availability and distance to the nearest infested house for the complete data set; presence of poultry, refuge availability, residential overcrowding and household educational level for the subset.

Under the null hypothesis that the models are indistinguishable, the Vuong’s test provided strong evidence of the superiority of the negative binomial model over the ZANB model (p < 0.001 for both data sets) and the ZINB model (p < 0.001 for both data sets). The ZIP model fitted the data poorly compared to the other models due to over-dispersion even in the positive part of the data. The following table shows the comparison between models.

Table. Log-likelihood, degrees of freedom and number of zeros for negative binomial regression and other alternative modeling approaches for bug abundance using the complete data set and the subset. The full models for count data included 10 (complete dataset) and 12 (subset) variables. For the binomial process, four covariates were included in all cases (complete data set: presence of poultry indoors, use of insecticide, refuge availability and distance to the nearest infested house; subset: presence of poultry, refuge availability, residential overcrowding and household educational level). ^a^ NB: negative binomial regression. ZANB (hurdle): zero-augmented negative binomial regression. ZINB: zero-inflated negative binomial regression. ZIP: zero-inflated Poisson regression.

| Data set | | Parameter | NB | ZANB (hurdle) | ZINB | ZIP |
| --- | --- | --- | --- | --- | --- | --- |
| Complete | Log-likelihood | | -429 | -423 | -421 | -1013 |
|  | Degrees of freedom | | 14 | 20 | 20 | 19 |
|  | Expected number of zeros^a^ | | 299 | 298 | 300 | 297 |
| Subset | Log-likelihood | | -275 | -274 | -272 | -498 |
|  | Degrees of freedom | | 16 | 20 | 20 | 19 |
|  | Expected number of zeros^b^ | | 214 | 215 | 216 | 215 |

^a^ A reduced number of variables was included as covariates in order to avoid over- parameterization, Covariates were included if significant (p < 0.05) in the zero part of the Hurdle model.

^b^ Observed number of zeros for the complete data set: 298; for the subset data: 215.

References

Vuong QH (1989). Likelihood ratio tests for model selection and non-nested hypotheses. Econometrica. 57: 307-333.

Zeileis A, Kleiber C, Jackman S (2008) Regression models for count data in R. J Stat Soft 27:1-25

Zuur AF, Ieno EN, Walker NJ, Saveliev AA, Smith GM (2009) Mixed effects models and extensions in ecology with R. Springer. 548 p
